# Redirecting full-length FLT1 expression towards its soluble isoform by targeting alternative polyadenylation promotes postischaemic angiogenesis

**DOI:** 10.1093/cvr/cvag198

**Published:** 2026-10-05

**Authors:** Maja Bundalo, Sandra Vorlova, Jessica Ulrich, Giuseppe Rizzo, Elke Butt, Hendrik Bartolomaeus, Hla Ali, Ruggero Barbieri, Leon Richter, Leonie Höna, Manuel Egg, Julian Bock, Sarah Schäfer, Núria Amézaga Solé, Annabelle Rosa, Clément Cochain, Andreas Beilhack, Wolfgang Kastenmüller, Erik Henke, Boris V Skryabin, Timofey S Rozhdestvensky, Moritz Wildgruber, Kari Alitalo, Kristina Lorenz, Michaela Kuhn, Alma Zernecke

**Affiliations:** Institute of Experimental Biomedicine, University Hospital Würzburg, Josef-Schneider-Str. 2, D16, Würzburg 97080, Germany; Institute of Experimental Biomedicine, University Hospital Würzburg, Josef-Schneider-Str. 2, D16, Würzburg 97080, Germany; Institute of Pharmacology and Toxicology, University of Würzburg, Versbacher Str. 9, Würzburg 97078, Germany; Institute of Experimental Biomedicine, University Hospital Würzburg, Josef-Schneider-Str. 2, D16, Würzburg 97080, Germany; Institute of Experimental Biomedicine, University Hospital Würzburg, Josef-Schneider-Str. 2, D16, Würzburg 97080, Germany; Institute of Experimental Biomedicine, University Hospital Würzburg, Josef-Schneider-Str. 2, D16, Würzburg 97080, Germany; Institute of Experimental Biomedicine, University Hospital Würzburg, Josef-Schneider-Str. 2, D16, Würzburg 97080, Germany; Institute of Experimental Biomedicine, University Hospital Würzburg, Josef-Schneider-Str. 2, D16, Würzburg 97080, Germany; Institute of Experimental Biomedicine, University Hospital Würzburg, Josef-Schneider-Str. 2, D16, Würzburg 97080, Germany; Institute of Experimental Biomedicine, University Hospital Würzburg, Josef-Schneider-Str. 2, D16, Würzburg 97080, Germany; Institute of Experimental Biomedicine, University Hospital Würzburg, Josef-Schneider-Str. 2, D16, Würzburg 97080, Germany; Institute of Experimental Biomedicine, University Hospital Würzburg, Josef-Schneider-Str. 2, D16, Würzburg 97080, Germany; Institute of Experimental Biomedicine, University Hospital Würzburg, Josef-Schneider-Str. 2, D16, Würzburg 97080, Germany; Institute of Experimental Biomedicine, University Hospital Würzburg, Josef-Schneider-Str. 2, D16, Würzburg 97080, Germany; Institute of Experimental Biomedicine, University Hospital Würzburg, Josef-Schneider-Str. 2, D16, Würzburg 97080, Germany; Institute of Experimental Biomedicine, University Hospital Würzburg, Josef-Schneider-Str. 2, D16, Würzburg 97080, Germany; PARCC, INSERM, Université Paris Cité, 56 rue Leblanc, 75015 Paris, France; Department of Internal Medicine II, Center for Experimental Molecular Medicine (ZEMM), Zinklesweg 10, University Hospital Würzburg, Würzburg 97078, Germany; Würzburg Institute of Systems Immunology, Max Planck Research Group, Julius-Maximilians University of Würzburg, Versbacher Straße 9, Würzburg 97078, Germany; Institute of Anatomy and Cell Biology, University of Würzburg, Koellikerstraße 6, Würzburg 97070, Germany; Department of Medicine, Core Facility Transgenic Animal and Genetic Engineering Models (TRAM), University of Münster, Von-Esmarch-Straße 56, Münster 48149, Germany; Department of Medicine, Core Facility Transgenic Animal and Genetic Engineering Models (TRAM), University of Münster, Von-Esmarch-Straße 56, Münster 48149, Germany; Department of Radiology, University Hospital, LMU Munich, Marchioninistraße 15, Munich 81377, Germany; Wihuri Research Institute, Biomedicum Helsinki, University of Helsinki, Haartmaninkatu 8, Helsinki 00290, Finland; Institute of Pharmacology and Toxicology, University of Würzburg, Versbacher Str. 9, Würzburg 97078, Germany; Department of Translational Research, Cardiovascular Pharmacology, Leibniz-Institut für Analytische Wissenschaften-ISAS-e.V., Bunsen-Kirchhoff-Str. 11, Dortmund 44139, Germany; Institute of Physiology, University of Würzburg, Röntgenring 9, Würzburg 97070, Germany; Institute of Experimental Biomedicine, University Hospital Würzburg, Josef-Schneider-Str. 2, D16, Würzburg 97080, Germany

**Keywords:** Angiogenesis, Myocardial infarction, Vasculogenesis, VEGF, Alternative polyadenylation

## Abstract

**Aims:**

Vascular endothelial growth factors (VEGFs) and their endothelial tyrosine kinase receptors are crucial mediators of vasculogenesis and angiogenesis, with FLT1 (VEGFR1) acting as a decoy receptor for VEGFs. Alternative polyadenylation (APA) of Flt1 transcripts produces a truncated mRNA transcript encoding soluble (s) FLT1, which contains only the extracellular ligand-binding domain. The relative roles of full-length (fl) FLT1 and sFLT1 in angiogenesis are incompletely understood. Alternative polyadenylation is an important post-transcriptional mechanism of gene regulation that is increasingly recognized to affect the expression of many genes related to cardiovascular diseases. However, there are no *in vivo* models available to target APA and to examine the physiological roles of gene isoforms.

**Methods and results:**

We here generated mice by introducing silent mutations into the *Flt1* exon 13/intron 13 5′ splice site through homology-directed repair with CRISPR/Cas9 gene editing. Embryonic development, postnatal retinal vascular development, and reparative angiogenesis were evaluated in models of hindlimb ischaemia and myocardial infarction, complemented by assessments of cardiac functional recovery and cell phenotypes through snRNAseq, as well as *in vitro* assays examining endothelial cell phenotype and function. The introduction of specific splice site mutations within Flt1 to activate APA led to a complete loss of flFLT1 and an increase in sFLT1 expression. Using this mouse model, we demonstrate that sFLT1 alone is sufficient for developmental vasculogenesis, whereas the specific loss of flFLT1 enhances reparative revascularization and angiogenesis and improves cardiac recovery after myocardial infarction.

**Conclusion:**

Our findings show that APA can be effectively targeted to modulate FLT1 isoform expression *in vivo*. Our mouse model may help to understand diseases driven by an imbalance in FLT1 isoform expression. Given the widespread presence of APA signals, our approach could furthermore serve as a model for studying the function of other truncated gene isoforms.

## Graphical Abstract

**Graphical Abstract:**
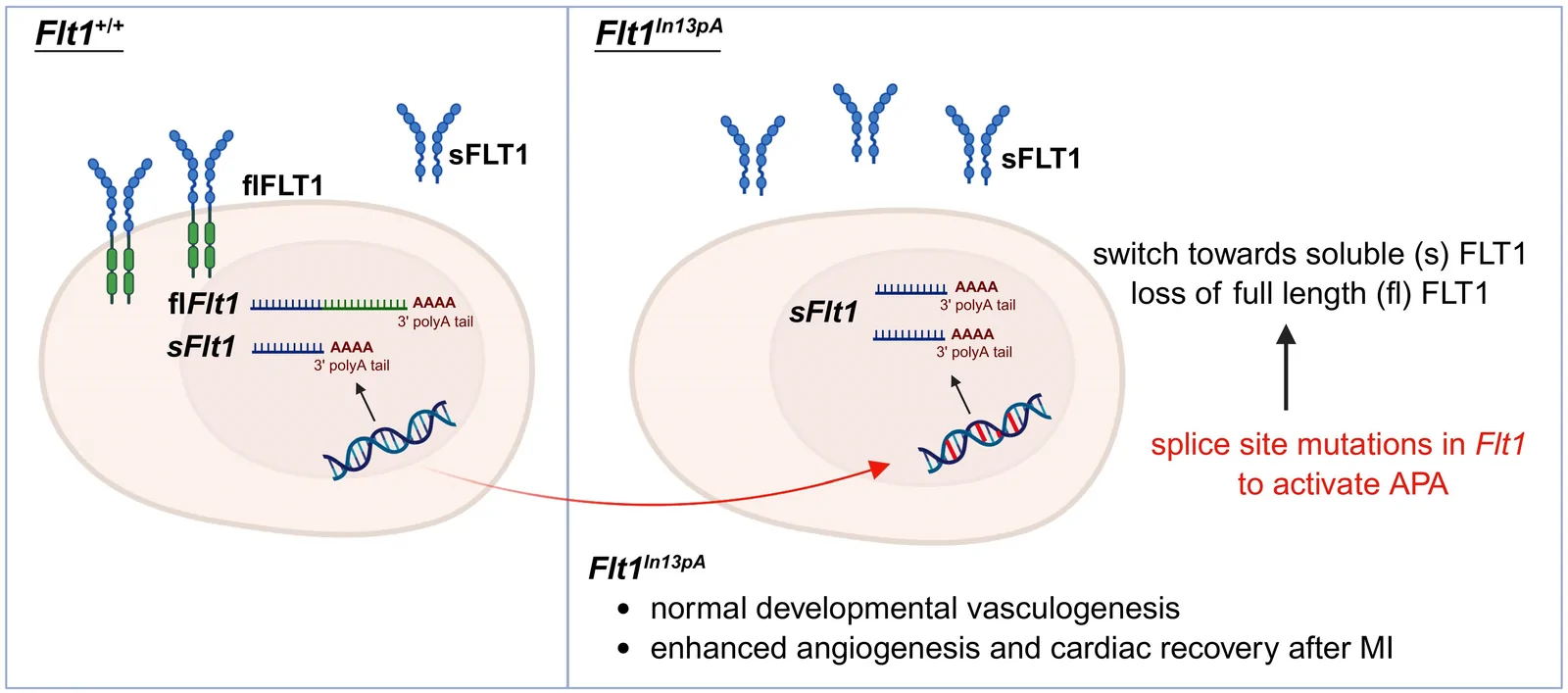

**Time of primary review: 43 days**

**See the editorial comment for this article ‘A novel strategy to protect ischaemic heart: driving FLT1 transcriptional switch’, by G. Bianchi and G. Lembo, https://doi.org/10.1093/cvr/cvag220.**

## 1. Introduction

Vascular endothelial growth factors (VEGFs) and their tyrosine kinase receptors VEGFR1/FLT1 and VEGFR2/KDR are key mediators of embryonic vasculogenesis and angiogenesis.^1,2^ Whereas VEGFR2 is primarily expressed in endothelial cells (ECs), regulating cell proliferation, chemotaxis, and cell survival,^3^ FLT1 is expressed in ECs, as well as in vascular smooth muscle cells and macrophages, and binds VEGF-A (VEGF) with a 10-folds higher affinity than VEGFR2.^4–6^ FLT1 is also an exclusive receptor for VEGF-B and placental growth factor (PlGF).^7,8^ sFLT1 retains and thereby sequesters the three growth factors but also interacts with full-length VEGF receptors, forming non-signalling homo- and heterodimers and reducing receptor kinase signalling.^9^

Mice deficient in FLT1 display excessive angioblast proliferation and disorganized vessels with an increased number of ECs.^6^ The cytoplasmic signalling function of full-length (fl) FLT1 appears dispensable for its decoy function, as mice with a targeted deletion of the tyrosine kinase domain of FLT1 are viable with a nearly normal vasculature.^10^ In contrast, mice lacking VEGFR2 die *in utero* due to severe deficiencies in vascular development.^11^ flFLT1 thus primarily functions as a sink for VEGF, indirectly regulating the signalling of VEGF through VEGFR2.

Vascular endothelial growth factor receptor 1 (VEGFR1, encoded by *Flt1*) is among the genes impacted by alternative polyadenylation (APA) in cardiovascular disease.^12^ The formation of mature mRNA requires extensive processing of newly transcribed RNA, including splicing and polyadenylation of the 3′ end of the pre-mRNA transcript. Alternative polyadenylation constitutes an additional layer of post-transcriptional gene regulation, profoundly influencing mRNA diversity, stability, and cellular function. Alternative polyadenylation in the last exon generates mRNA isoforms that encode the same protein but with a different 3′ untranslated region (UTR). The activation of intronic or coding region APA signals can lead to the generation of truncated protein isoforms. Notably, approximately 70–75% of human genes contain APA signals, and a growing number of genes are emerging to be affected by APA also in cardiovascular diseases, including genes relevant to cardiac hypertrophy and heart failure, as well as myocardial infarction (MI).^12^ Most of these studies, however, focused on APA events within the 3′ UTR. Much less is known about intronic or coding region APA and the function of such shortened gene isoforms. Moreover, there are no preclinical models available that target APA to study their physiological generation and function *in vivo*.

The activation of an APA signal within intron 13 of the *Flt1* mRNA leads to the production of a truncated but stable mRNA. This shortened *Flt1* transcript lacks the transmembrane spanning and intracellular regions, resulting in an isoform known as soluble FLT1 (sFLT1). High circulating levels of sFLT1 are found in patients with acute MI, are an independent predictor for the development of acute severe heart failure,^13^ and were associated with an increased risk of human cardiovascular disease events in the Dallas Heart Study.^14^ The mechanisms behind these correlations and the relative role of sFLT1 vs. flFLT1 in the context of tissue injury, however, are unknown.

Here, we set out to investigate if we could shift the balance between flFLT1 and sFLT1 isoform expression *in vivo* by site-specifically activating the APA signal in intron 13. We demonstrate for the first time that targeting APA can be harnessed to redirect gene isoform expression *in vivo*. Mice with a mutated 5′ splice site in intron 13 indeed showed a complete loss of the transmembrane flFLT1 receptor and a concomitant switch towards expression of the truncated sFLT1 isoform only. By completely switching the endogenous expression from *flFlt1* towards *sFlt1* in our novel mouse model, we furthermore pinpointed that sFLT1 (in the absence of flFLT1) is sufficient for normal developmental vasculogenesis and postnatal angiogenesis, whereas flFLT1 constrains postischaemic angiogenesis in the adult.

## 2. Methods

### 2.1 RNA extraction, reverse transcription, and qPCR

Total RNA was isolated from various tissues, BMDM, and mouse lung endothelial cells using the NucleoSpin RNA kit (Macherey Nagel #740955) or Trizol according to the manufacturer’s instructions. RNA was transcribed using the First Strand cDNA Synthesis Kit (Thermo Scientific, K1612). Polymerase chain reaction (PCR) and quantitative polymerase chain reaction (qPCR) were performed using two reverse primers for *sFlt1* and *flFlt1* (TTAATGTTTGACATGACTTTGTGTG and CGGTTCTTGTTGTATTTTGTGGTTG) and a common forward primer (GCCTTCAATAAAATAGGGACTGTGG). For qPCR, total *Flt1* was amplified using forward primer CACCACGGAGCTCAATACGA and reverse primer CTTCACGCGACAGGTGTAGA. Quantitative polymerase chain reaction was performed using a GeneQuant Studio 6 Real-Time PCR System and SybrGreen PCR Mix. Results are expressed as 2^−ΔΔCt^. For absolute qPCR quantification, gBlocks were used as standards. Sequences were as follows:

*s*/*tFlt1* tgcatgatctacgtgcgtcacatgcagtacGCCTTCAATAAAATAGGGACTGTGGAAAGAAACATAAAATTTTATGTCACAGATGTGCCGAATGGCTTTCACGTTTCCTTGGAAAAGATGCCAGCCGAAGGAGAGGACCTGAAACTGTCCTGTGTGGTCAATAAATTCCTGTACAGAGACATTACCTGGATTCTGCTACGGACAGTTAACAACAGAACCATGCACCATAGTATCAGCAAGCAAAAAATGGCCACCACTCAAGATTACTCCATCACTCTGAACCTTGTCATCAAGAACGTGTCTCTAGAAGACTCGGGCACCTATGCGTGCAGAGCCAGGAACATATACACAGGGGAAGACATCCTTCGGAAGACAGAAGTTCTCGTTAGAGGTGAGCACTGCGGCAAAAAGGCCATTTTCTCTCGGATCTCCAAATTTAAAAGCAGGAGGAATGATTGTACCACACAAAGTCATGTCAAACATTAAaccaatggctttccgagatgCACCACGGAGCTCAATACGAGGGTGCAAATGAGCTGGAATTACCCTGGTAAAGCAACTAAGAGAGCATCTATAAGGCAGCGGATTGACCGGAGCCATTCCCACAACAATGTGTTCCACAGTGTTCTTAAGATCAACAATGTGGAGAGCCGAGACAAGGGGCTCTACACCTGTCGCGTGAAGcactagctcagattcagtagaccgctgttg

*flFlt1*

tgcatgatctacgtgcgtcacatgcagtacGAGCGATGTGGAAGGAGACGAGGATTACAGTGAGATCTCCAAGCAGCCCCTCACCATGGAAGACCTGATTTCCTACAGTTTCCAAGTGGCCAGAGGCATGGAGTTTCTGTCCTCCAGAAAGTGCATTCATCGGGACCTGGCAGCGAGAAACATCCTTTTATCTGAGAACAATGTGGTGAAGATTTGCGACTTTGGCCTGGCCCGGGATATTTATAAGAACCCTGATTATGTGAGGAGAGGAGATACTCGACTTCCCCTAAAATGGATGGCTCCTGAATCCATCTTTGACAAGGTCTACAGCACCAAGAGCGATcactagctcagattcagtagaccgctgttg

*flFlt1* was amplified using forward primer GAGCGATGTGGAAGGAGACGAG and reverse primer ATCGCTCTTGGTGCTGTAGACC.

### 2.2 Morpholino treatment

Mouse *Flt1* antisense (MoVE) oligonucleotide sequences were 5′-TTTTTGCCGCAGTGCTCACCTCTAA-3′. Oligonucleotides were transfected into 4 × 10^4^ cells using Endoporter (Gene Tools) according to the manufacturer’s instructions to achieve the desired final doses as indicated.

### 2.3 Generation of *Flt1^In13pA^* mice

*Flt1* ^*In13pA*^ mouse line was generated by direct oocyte microinjections using the CRISPR/Cas9 components together with the single-stranded oligo-deoxyribonucleotide (sODN) donor template (FLT1mutTEMPL) with the subsequent surgical embryo transfer. *Flt1*^*In13pA*^ founder mice were backcrossed into the C57BL/6 background for ten generations.

### 2.4 Donor DNA template preparation

Donor DNA template FLT1mutTEMPL: (TGCAGAGCCAGGAACATATACACAGGGGAAGACATCCTTCGGAAGACAGAAGTTCTCGTTcGcGGcGAaCACTGCGGCAAAAAGGCCATTTTCTCTCGGATCTCCAAATTTAAAAGCAGG) was chemically synthesized [MWG, Eurofins].

### 2.5 Mouse oocyte microinjections

For the preparation of CRISPR/Cas9 microinjection solution, commercially synthesized FLT1_crR1 with target sequence CTCGTTAGAGGTGAGCACTG, together with the tracrRNA and Cas9 protein [Integrated DNA Technologies (IDT), USA], were mixed as follows: 50 pmol of crRNA were mixed with 50 pmol of tracrRNA in 10 mM potassium acetate and 3 mM Hepes (pH 7.5) buffer and incubated at 95°C for 1 min, followed by cooling to room temperature. The annealed crRNA/tracrRNA complex was mixed with Cas9 mRNA, Cas9 protein, and FLT1mutTEMPL sODN. The final concentrations of CRISPR/Cas9 components in 0.6 mM Hepes (pH 7.5) and 2 mM potassium acetate microinjection buffer were as follows: crRNA (1 pmol/µL), tracrRNA (1 pmol/µL), Cas9 mRNA (10 ng/µL), Cas9 protein (25 ng/µL), and FLT1mutTEMPL sODN donor template (0.04 pmol/µL). The final injection solution was filtered through Millipore centrifugal columns and spun at 20 000 *g* for 10 min at +4^o^C. Microinjections were performed in B6D2F1 (hybrid between C57BL/6J and DBA strains) *in vitro* fertilized (IVF) one-cell oocytes. For the IVF, oocytes were removed from the oviducts of superovulated B6D2F1 female mice in 90 µL of 0.25 mM GSH in mHTF media under the mineral oil. In parallel, *cauda epididymis* and *vas deferens* (*ductus deferens*) of the C57BL6 male mouse were transferred to 120 µL of TYH MBCD media and mixed. Ten microliters of sperm solution were transferred to fresh 90 µL of TYH MBCD medium and kept for 1 h at 37°C, 5% CO_2_. Ten microliters of the diluted sperm solution were added to isolated oocytes and placed in an incubator at 37°C and 5% CO_2_ for 3 h. After IVF, zygotes were washed four times with 120 µL of warm mHTF medium and incubated for 3 h at 37°C and 5% CO_2_. Microinjections were performed in M2 media, using the Transjector 5246 (Eppendorf) and Narishige NT-88NE micromanipulators attached to a Nikon Diaphot 300 inverted microscope. Zygotes that survived microinjections were incubated in KSOM medium overnight at 37°C and 5% CO_2_. The next day, two-cell embryos were transferred to the oviducts of pseudopregnant CD1 foster mice and carried to term. Mice were anaesthetized by intraperitoneal injection of 10% ketamine and 2% xylazine, corresponding to a final concentration of ketamine/xylazine 83/16 mg/kg of body weight. Positively targeted F0 and F1 animals were identified by qPCR and sequencing analysis of genomic DNA isolated from ear biopsies.

### 2.6 *Flt1* ^*In13pA*^ mouse genotyping

TaqMan qPCR analysis was applied for genotyping of *Flt1*^*In13pA*^ mice. The qPCR reaction was performed using 100 ng of genomic DNA samples, using FLT1_qPCRd1 GGAAGACATCCTTCGGAAGAC and FLT1_qPCRr1 AATTTGGAGATCCGAGAGAAAA primers in combination with dual-labelled LNA probes 5′-6FAM/3′-IBFQ: (IDT, USA): Flt1wt AGAGGTGAGC (wild type) or Flt1mut CGCGGCGAAC (mutant). The position of LNA nucleotides in the probe is underlined. All qPCR reactions were performed in a total volume of 20 μL containing 2 μL of genomic DNA (∼100 ng), 10 μL of 2×TaqMan Master Mix (Roche), 0.1 μM TaqMan LNA probe, and 0.1 μM of each primer. Polymerase chain reaction amplification was performed as follows: enzyme activation at 95°C for 5 min [ramp rate (RR) 4.4], with subsequent 50 cycles of qPCR [95°C, 10 s (RR 4.4); 60°C, 30 s (RR 2.2)] and final cooling cycle [40°C, 10 s (RR 2.2)]. Polymerase chain reaction products from qPCR-positive *Flt1*^*In13pA*^ mice were confirmed by sequencing.

### 2.7 Mouse experiments

All animal studies and numbers of animals used conform to the Directive 2010/63/EU of the European Parliament and have been approved by the appropriate local authorities (Regierung von Unterfranken, Würzburg, Germany, Akt.-Z 55.2.2-2532-2-1272 and Akt.-Z 55.2.2-2532-2-938). Mice were killed by an overdose of isoflurane anaesthesia (inhalation, 5 Vol.-%) and cervical dislocation.

#### 2.7.1 Myocardial infarction

Myocardial infarction was induced in 8- to 12-week-old male C57BL/6 mice by permanent ligation of the left descending coronary artery. Mice received 0.1 mg/kg buprenorphine intraperitoneally as preemptive analgesia (Bupresol vet. multidose 0.3 mg/mL) before surgery, and anaesthesia was induced by inhalation of 4 Vol.-% isoflurane (Isofluran CP, 1 mL/mL). Mice under deep anaesthesia were intubated with an endotracheal cannula and placed under mechanical ventilation (CWE INC. SAR-830-AP; 130 respirations/min, peak pressure max. 18 cm H2O) on a heating pad to maintain body temperature. 1.5–2.0% isoflurane was used to maintain anaesthesia during surgery. The heart was exposed via a fourth intercostal thoracotomy. The left descending coronary artery was visualized and ligated with a 7/0 non-resorbable nylon suture. The thorax was closed with three separated 6/0 non-resorbable nylon sutures and the skin with a continuous 6/0 non-resorbable nylon suture. Buprenorphine (0.1 mg/kg) was administered intraperitoneally 4-6 h after the surgery and twice daily for 2 days following surgery, combined with overnight *ad libitum* oral supplementation of Medigel Sucralose containing buprenorphine (5 µg/mL). Exclusion criteria related to general animal health had been defined upon pre-registration of the experiments for approval by local authorities. Briefly, animal well-being was controlled at regular intervals. The well-being of the animals was monitored using a cumulative point system (score sheet), with a predefined threshold associated with early termination of experiments and humane euthanasia. For MI studies, mice dying after MI before the planned sacrifice date were excluded from analysis after 28 days. At sacrifice, specific exclusion criteria related to the size of the infarct were applied: mice with the absence of a visible infarct or mice showing small infarcts (below ∼30% of the left ventricle, as macroscopically determined by the experimentator after sacrifice) were excluded from analysis; applying these criteria, we excluded two mice from the study.

For the analysis of cardiac function, mice were anaesthetized by a single intraperitoneal injection of pentobarbital (20–35 mg/kg) before examination using a Vevo2100 (VisualSonics) high-resolution imaging device and a 30-MHz probe.^15^ Briefly, animals were monitored by surface ECG using limb electrodes and body temperature (37°C warming plate, anal probe). Mice were shaved and fixed on the warmed ECG plate in a prone position. B-mode movies of the parasternal long axis (PSLAX) and an orthogonal short axis at the proximal level of the papillary muscle were acquired. For the analysis of end-diastolic and end-systolic volumes, the endocardium of the left ventricle was traced at both diastole and systole. An integrated software tool (Simpson) was used to analyse ventricular volumes and FAC. Ejection fraction (EF) was calculated using the formula EF = ((EDV—ESV)/EDV) × 100, where EDV is end-diastolic volume. Mid-ventricular M-mode was used to determine the systolic (s) and diastolic (d) left ventricular internal diameter (LVID) and to calculate fractional shortening (FS %). Mice with heart rates below 430 beats per minute were excluded from the analyses.

#### 2.7.2 Hindlimb ischaemia

Twelve-week-old *Flt1^+/+^* and *Flt1^In13pA^* mice received a single intraperitoneal injection of buprenorphine (0.1 mg/kg) as analgesia (Bupresol vet. multidose 0.3 mg/mL) and were anaesthetized with inhalation of 3-5 Vol.-% isoflurane (Isofluran CP, 1 mL/mL). During surgery, 1.5-2.0% isoflurane was used to maintain anesthesia, and mice were placed on 37°C heated pads. Hair was removed from the hindquarters, and the femoral artery was exposed and isolated from the femoral vein and nerve. Unilateral hindlimb ischaemia was induced by partial excision of the left femoral artery. After 14 days, mice were sacrificed, and hindlimb muscles were excised for further analysis.

#### 2.7.3 Air pouch model

Mice were anaesthetized with 2 Vol.-% Isofluran, and 5 mL of sterile air was subcutaneously injected in the intrascapular area of the back. On day 3, an additional 3 mL of sterile air was injected. On day 5, sterile PBS or PBS + 50 ng/mL VEGF (Peprotech #450-32) was injected into the air pouch, and 24 h later, exudates were collected. The number of leukocytes in the exudate was counted using a Neubauer counting chamber, and a flow cytometric analysis was performed.

### 2.8 Single-nuclei RNA sequencing

Left ventricular cell nuclei were isolated and loaded into the 10X Genomics Chromium system for library preparation. After passing library quality control, sequencing is performed using a second-generation sequencing platform, with a sequencing strategy of PE150. The libraries were sequenced on the NovaSeq6000 sequencer (Illumina). Quality control analysis of the transcript sequences for each sample is performed using the FastQC software. Raw reads were processed using CellRanger. Reads were aligned to the reference genome (GRCm38) using STAR. Filtering, unsupervised clustering, differential expression, and additional analysis were done using R (4.3.1) software. Quality control filtering was performed with exclusion of nuclei with mitochondrial transcript content higher than 5% and 5000 < nFeature_RNA < 3000. For matrix analysis, we used the standard Seurat workflow. Matrix was log-normalized and scaled. Integrative analysis was performed to correct for potential batch effects and technical differences in sequencing. Clustering was performed using the ‘FindClusters’ with a resolution parameter set to 0.15. Endothelial cells were then extracted and reclustered with the ‘FindClusters’ function. A gene expression analysis was done by pseudo-bulk RNA-seq analysis using the DESeq2 package for R with an alpha value of 0.1. Gene enrichment analysis have been performed using the ‘EnrichGO’ function of the ClusterProfiler. Mouse matrisome scores were downloaded from the ‘The Matrisome Project’ (https://doi.org/10.1074/mcp.M111.014647) and applied to the fibroblast cluster using the ‘AddModuleScore’ function. Statistics were calculated using the t.test in the ‘ViolinPlot2’ function from the ‘SeuratExtend’ package.

### 2.9 ELISA

Serum and cell culture supernatant concentrations of sFLT1 were measured by ELISA according to the manufacturer’s instructions (R&D, #DY471). For measurements of sFLT1 in EC culture supernatants, 4 × 10^4^ mouse lung endothelial cells isolated from *Flt1^+/+^* and *Flt1^In13pA^* mice were plated on 24-well plates in complete EC media. After 24 h, when the cells reached confluence, the medium was changed to 1% FCS media. Cells were incubated for an additional 28 h, and supernatant was collected and centrifuged at 400 g for 5 min. Serum PlGF concentrations were measured by ELISA as specified by the manufacturer (Proteintech, #KE10084).

### 2.10 3′ RACE experiments

3′ RACE reactions were performed on RNA extracted from mouse liver tissue, and the RACE experiments were done according to the manufacturer’s instructions (3′ RACE System for Rapid Amplification of cDNA Ends, Invitrogen).

### 2.11 Mouse lung endothelial cell isolation

Mouse lung endothelial cells were isolated from the lungs of 6–8-week-old mice, as described.^16^ Three millilitres of cold PBS were injected via the heart to flush the lung of blood cells. Lungs were removed, cut into small pieces, and incubated with dispase II (25 U) for 1 h at 37°C, with vigorous shaking at 350 rpm. Cell suspension was filtered through a 70 µm strainer and washed with 50 mL of DMEM-F12 with 10% FCS, 100 U/mL penicillin, and 100 μg/mL streptomycin. Cells were pelleted by centrifugation for 5 min at 300 g and plated in gelatin-coated flasks in complete EC media—DMEM-F12 with 20% FCS, 100 U/mL penicillin, and 100 μg/mL streptomycin with the addition of ECGF (12 µg/mL, PeloBiotech #PB-ECGF-1 Heparin). After reaching confluence, cells were detached by accutase (Sigma Aldrich #A6964), and endothelial cells were selected using CD31 murine MicroBeads according to the manufacturer’s instructions (Miltenyi, #130-097-418). Cells were further cultivated on gelatin-coated plates in complete EC media.

### 2.12 Tube formation assay

Forty-eight-well plates were covered with 50 µL of Geltrex previously thawed overnight at 4°C (Gibco # A1413201) and incubated for 30 min at 37°C. 3 × 10^4^ mouse lung endothelial cells isolated from *Flt1^+/+^* and *Flt1^In13pA^* mice were plated per well in complete EC media and incubated for 24 h at 37°C and 5% CO_2_. Images were taken by a Nikon Eclipse TS2 microscope, and the number of meshes was counted per microscopic field. To investigate the effect of exogenously added VEGF, 3 × 10^4^ mouse lung endothelial cells isolated from *Flt1^+/+^* and *Flt1^In13pA^* mice were plated in Geltrex-coated 48-well plates in supernatant collected from *Flt1^+/+^* and *Flt1^In13pA^* mouse lung endothelial cell cultures containing sFLT1 (*Flt1^+/+^* supernatant: 2000pg/mL sFlt1; *Flt1^In13pA^* 5242 pg/mL sFLT1) in DMEM-F12 with 5% FCS, 100 U/mL penicillin, and 100 μg/mL streptomycin. Cells were treated with 50 ng/mL of recombinant murine VEGF (Peprotech #450-32). After 24 h of incubation, images were recorded by a Nikon Eclipse T2 microscope. To investigate if sFLT1 produced by *Flt1^In13pA^* EC is able to repress VEGF-induced angiogenesis, 2 × 10^4^ SVEC cells^17^ were plated on Geltrex-coated 48-well plates in DMEM-F12 (0.5% FCS) or in supernatant collected from *Flt1^+/+^* and *Flt1^In13pA^* mouse lung endothelial cell cultures with the addition of 50 ng/mL of recombinant murine VEGF. Cells were incubated for 24 h at 37°C and 5% CO_2_. Images were taken after 2, 4, 6, and 24 h by a Nikon Eclipse TS2 microscope. The number of tubes was counted per microscopic field.

### 2.13 Endothelial cell proliferation

2 × 10^3^ mouse lung endothelial cells were plated in gelatin-coated 48-well plates in complete EC media and incubated for 48 h at 37°C and 5% CO2. Cells were then washed with PBS, detached by accutase, centrifuged, and resuspended in 20 µL of PBS. 10 µL of cell suspension was diluted in 90 µL of trypan blue solution (Sigma #T8154), and the number of live cells was counted using a hemocytometer.

### 2.14 *In vitro* macrophage migration assay

Bone marrow-derived macrophages were flushed from the mouse femur and tibia, filtered through a 70 µm nylon mesh, and plated in 10 cm dishes at a density of 2 × 10^6^/mL in 10 mL. Cells were differentiated into macrophages in medium containing RPMI1640, 15% L929-conditioned medium, 10% foetal bovine serum (heat-inactivated at 56°C/45 min), 50 µM β-mercaptoethanol (Gibco #31350-010), 100 U/mL penicillin, and 100 μg/mL streptomycin for 7 days. Before the migration assay, cells were starved for 4 h in RPMI medium containing 0.5% FCS. Accutase was used to harvest the cells, and 10^5^ cells were transferred to the transwell inserts (100 µL of cell suspension in 0.5% FCS medium). Cells were allowed to settle down for 30 min before adding attractants to the receiver well—50 ng/mL VEGF (Peprotech #450-32) or 100 ng/mL CCL2 (Peprotech #250-10). Cells were incubated for 4 h at 37°C and 5% CO_2_. After migration, 4% PFA was used to fix the cells, and they were stained with 1% crystal violet. Transwell inserts were washed in distilled water to remove excess crystal violet, and non-migrated cells on top of the membrane were removed with cotton swabs. Images were taken by a Leica DM4000 microscope. Macrophage migration index was calculated as the number of cells migrated towards the attractant divided by the number of spontaneously migrated macrophages in the negative control.

### 2.15 Immunostainings of retinal endothelial cells

Eyes were fixed in 4% PFA for 15 min. The retinal cups were dissected and fixed for an additional 2 h at 4°C. Retinal EC were then stained overnight with FITC-conjugated isolectin B4 from Bandeirae simplicifolia (0.04 mg/mL; Sigma-Aldrich #L9381) in Pblec solution (1 mM MgCl2, 1 mM CaCl2, 0.1 mM MnCl2, 1% Triton X-100 in PBS) at 4°C. The retinal cups were washed in PBS, cut into quadrants, and flat-mounted on microscopic slides with Mowiol solution (Dianova). Images were taken by a fluorescent microscope (Olympus, Hamburg, Germany). To study physiological vascular development at P5 and P7, the vascular and entire retinal areas of each quadrant were analysed using ImageJ and Angiotool software.

### 2.16 3′mRNA sequencing and analysis

The total RNA from mouse lung endothelial cells was analysed by 3′ mRNA sequencing with QuantSeq 3′ mRNA-Seq Library Prep Kit REV for Illumina (Lexogen #016.24). Library preparation and sequencing were done according to the manufacturer’s instructions. Samples were sequenced in triplicate. FASTQ files went through pre-processing according to Lexogen’s instructions. Briefly, reads were trimmed with Cutadapt^18^ to remove residual technical sequences. The trimmed reads were then aligned against the mouse genome (Gencode M29) with STAR.^19^ PolyA site read counts for Flt1 were obtained using the Samtools^20^ view command with a filter for mapping quality lower than 30 applied. Genomic coordinates were used to determine the intervals where reads were counted. These were obtained from the PolyASite^21^ database for known sites and determined as a 10 bps interval around putative unannotated polyA sites. PolyA sites were examined by observing the sequence composition and read coverage with the IGV^22^ genome viewer. Proportions (in percentages) were then calculated on the read counts sum for all valid isoforms. RNA sequencing data were analysed by R software, including principal component analysis, DESeq2 analysis, and Gene Set Enrichment Analysis (GSEA). Downstream statistical analysis of differential gene expression was conducted using the DESeq2 Bioconductor package (*P* adjusted ≤ 0.001, log2cutoff ≤ 0.5). Angiogenesis-related genes presented in the heatmap were obtained from the molecular signature database (mouse gene set—Hallmark_Angiogenesis, GSEA, www.gsea-msigdb.org) and the Gene Ontology Term angiogenesis (GO:0001525).

### 2.17 Flow cytometry

For air pouch analyses, exudates were collected and centrifuged. Cells were stained for surface markers with specific antibodies: anti-CD45 (Biolegend #103128, dilution 1:400), anti-CD11b (Biolegend #101237, dilution 1:400), anti-CD115 (Invitrogen #17115280, dilution 1:300), anti-Ly6G (BD #560603, dilution 1:400), anti-Ly6C (Biolegend #128033, dilution 1:400), anti-CCR2 (Biolegend #150609, dilution 1:20), anti-F4/80 (Biolegend #123114, dilution 1:100), anti-CD64 (Biolegend #139316, dilution 1:100), and anti-MHCII (Biolegend #BV785, dilution 1:100). Probes were analysed using a FACSCelesta and FlowJo 10.0 software (BD Biosciences). Endothelial cell purity was analysed by staining of surface markers anti-CD31 (Invitrogen #11-0311-82, dilution 1:300) and anti-CD144 (Miltenyi #130-128-207, dilution 1:10). Three days after MI, hearts were removed and enzymatically digested in RPMI medium containing 450 U/mL collagenase I (Sigma-Aldrich #C0130), 125 U/mL collagenase XI (Sigma-Aldrich #C7657), and 60 U/mL hyaluronidase (Sigma-Aldrich #H3506) for 1 h at 37°C with agitation. Thereafter, the digested material was passed through a 70 µm cell strainer. Unspecific binding to Fc receptors was blocked by incubation of the cell suspension with anti-CD16/32 (Biolegend #101302, dilution 1:50) at 4°C for 10 min. After pelleting, cells were stained with Fixable Viability Dye (ThermoFisher, 1:1000) and fluorochrome conjugated antibodies against anti-CD45 FITC (Invitrogen # 11-0451-82, dilution 1:300), anti-CD11b PercpCy5.5 (BioLegend #101228, dilution 1:400), anti-Ly6C BV510 (BioLegend #128033, dilution 1:300), anti-Ly6G Pacific Blue (BioLegend #127612, dilution 1:100), CD115-APC (Invitrogen #17-1152-82, dilution 1:300), anti-F4/80 PeCy7 (Biolegend #123114, dilution 1:300), anti-TIM4 (BD Bioscience #564147, dilution 1:100), and anti-MHCII BV785 (Biolegend, #107645, dilution 1:300) at 4°C for 30 min. Results were acquired using a FACS Celesta (BD Biosciences) and analysed with FlowJo 10.5.3.

### 2.18 Histology and immunohistochemistry

Hindlimb muscles and hearts were fixed overnight in 4% para­formaldehyde and embedded in paraffin. 5 µm sections were used for immunohistochemistry. Paraffin was removed from sections by consecutive washes with xylene and rehydration in decreasing ethanol %. Citrate antigen retrieval and 1 h of blocking with anti-mouse CD16/32 (Biolegend #101302, dilution 1:50) were performed before staining. Endothelial cells were stained with anti-CD31 antibody (R&D #AF3628, dilution 1:70) and macrophages with Mac2 antibody (Cederlane #CL8942AP, dilution 1:500). The following day, slides were washed in PBS 3 × 5 min and then stained with the secondary antibody Goat anti-rabbit AlexaFluor555 (Invitrogen #A21429, working concentration 2 µg/µL) or Goat anti-rat Alexa488 (Thermofisher #A-11006, working concentration 4 µg/µL) in PBS for 1 h. To visualize cardiomyocytes, Rhodamine (Vector Lab. # RL-1022) was added together with the secondary antibody. After washing in PBS (3 × 5 min), nuclei were counterstained with DAPI (Vectashield #H-1200). Images were taken using a Leica DM4000 microscope and analysed using Diskus software.

### 2.19 Protein lysate preparation and Western blotting

8 × 10^4^ mouse lung endothelial cells isolated from *Flt1^+/+^* and *Flt1^In13pA^* mice were plated on gelatin-covered 12-well plates. After 24 h, cells were washed three times with ice-cold PBS and then lysed in ice-cold RIPA buffer (20 mM Tris pH 7.4, 150 mM NaCl, 1% Na-Deoxycholat, 1% TritonX 100, 0.1% SDS, 10 mM EDTA, 0.5 mM Na-Orthovanadat, 10 mM Na-Pyrophosphat, 0.5 mM p-Nitrophenylphosphate). Mouse tissues were minced on dry ice and lysed in ice-cold RIPA buffer. Samples were vortexed and kept on ice for 30 min. Thereafter, we proceeded with sonication for 1 min at a power of 180 W (10 s sonication/10 s rest for each cycle). Samples were centrifuged at 10 000×g for 20 min at 4°C to pellet cell debris, and the supernatant was transferred to a fresh microfuge tube. Total protein concentration was determined by BCA kit (Thermo Fisher #23225). For Western blotting, EC lysates were mixed with 2X sodium dodecyl sulphate (SDS) sample buffer to a final dilution of 1X and heated at 95°C for 5 min, and 40 µg of protein lysate was separated by 8% SDS polyacrylamide gel. We loaded 10 µg of heart and kidney protein lysate and 45 µg of liver protein lysate on 8% SDS polyacrylamide gel. Electrophoresis was done following standard protocols, and probes were transferred to nitrocellulose membranes. Membranes were blocked with 3% BSA in Tris-buffered saline with 0.1% Tween 20 (TBST) for 3 h followed by overnight incubation at 4°C with primary antibodies: anti-VEGFR1 (R&D # AF471, dilution 1:500), anti-phosphoY1175-VEGFR2 (Biorbyt #0rb256644, dilution 1:500), anti- VEGFR2 (Cell Signaling #2479, dilution 1:1000), Vinculin (Santa Cruz #sc-5573, dilution 1:500), and anti-Actin (Santa Cruz #sc-47778, dilution 1:1000). Membranes were then washed three times in TBST and probed with horseradish peroxidase-conjugated goat anti-rabbit or goat anti-mouse antibody (Biorad, Feldkirchen, Germany). Visualization was done using enhanced chemiluminescence reagent (GE Healthcare, München, Germany), and chemiluminescence images were taken using the Amersham Imager 600 (GE Healthcare, München, Germany).

### 2.20 Statistics

Results are presented as mean ± SD and were analysed using Prism9 Software (GraphPad, Inc, La Jolla, CA). The unpaired Student’s *t*-test was used for two-group comparisons with normally distributed data. For multiple group comparisons of normally distributed data, the two-way ANOVA followed by the multiple-comparison Tukey’s *post hoc* test was used. *P* < 0.05 was considered statistically significant. The individual sample sizes for each set of data are provided in the figures and their legends.

## 3. Results

### 3.1 Myocardial injury differentially affects tissue expression of soluble and full-length *Flt1*

U1 small nuclear ribonucleoprotein (U1 snRNP) suppresses premature cleavage and polyadenylation of nascent RNA polymerase II transcripts at cryptic polyA signals, a process termed telescripting. Alternative *Flt1* pre-mRNA processing involves retention of intron 13 and activation of the intronic APA signal to produce *sFlt1* (*Figure 1A*).^23^ We first measured the absolute mRNA expression levels of both *sFlt1* and *flFlt1* isoforms, and the highest levels were seen in mouse hearts and kidneys (*Figure 1B*).

**Figure 1:**
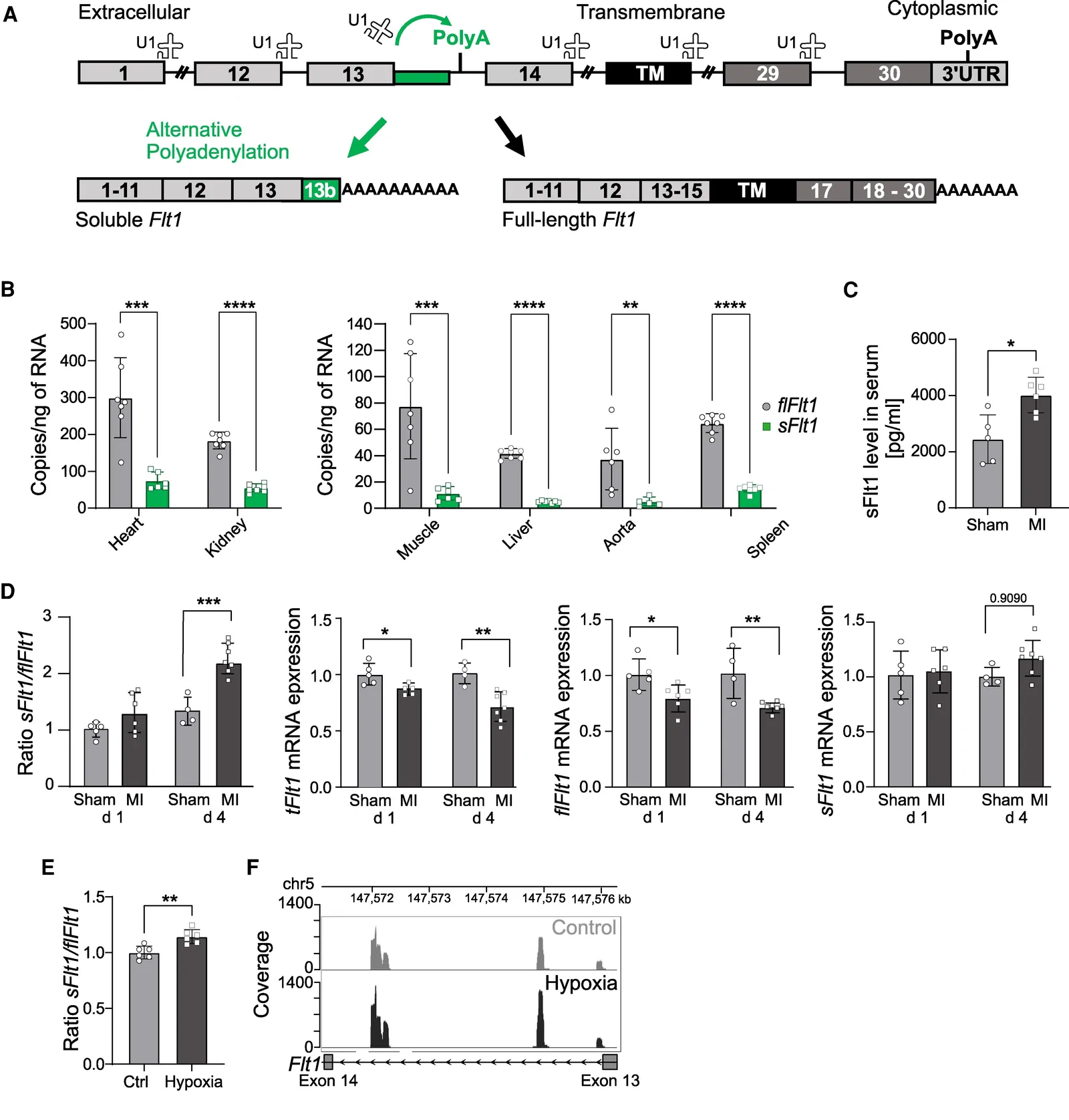
Expression of *flFlt1* and *sFlt1* mRNA is differentially regulated under hypoxia and in response to myocardial injury. (*A*) Scheme of *Flt1* genomic structure. Interfering with telescripting by impeding binding of U1 snRNP to the 5’ splice site in intron 13 leads to the activation of an alternative cleavage–polyadenylation (polyA) signal within intron 13, resulting in a stable truncated transcript with partial retention of intron 13. (*B*) Absolute number of mRNA copies of *flFlt1* and *sFlt1* in indicated *Flt1*^+/+^ tissues (*n* = 5–7 mice), analysed using gBlocks as a standard. (*C*) Serum level of sFLT1 in serum of mice at Day 1 after MI (*n* = 5 mice) compared to sham controls (*n* = 6 mice). (*D*) qPCR analysis and *sFlt1*/*flFlt1* ratio and *tFlt1*, *flFlt1*, and *sFlt1* expression in the infarcted myocardium compared to tissue from sham controls (*n* = 4–7 mice per group). (*E*) qPCR analysis and *sFlt1*/*flFlt1* ratio in lung endothelial cells exposed to hypoxia over 24 h (*n* = 6 experiments performed with pooled endothelial cells isolated from *n* = 8 mice). (*F*) Differential *Flt1* Intron 13 polyA site (PAS) usage in control and hypoxia-exposed endothelial cells. Data represent mean ± SD. Results were analysed by two-tailed, unpaired Student’s t-test. *P < 0.05, **P < 0.01, ***P < 0.001, ****P < 0.0001. *tFlt1*, total *Flt1*; *flFlt1*, full length *Flt1*; *sFlt1*, soluble *Flt1*; MI, myocardial infarction.

Patients with MI exhibit elevated serum levels of sFLT1.^13^ Consistent with these clinical observations, mice subjected to MI induced by LAD ligation showed increased serum sFLT1 levels (*Figure 1C*). In the infarcted myocardium, an increase in the ratio of *sFlt1* to *flFlt1* mRNA expression was observed, due to reduced *flFlt1* and overall *Flt1* levels and modestly increased *sFlt1* expression (*Figure 1D*). Also, in primary murine EC rendered hypoxic, as confirmed by the induction of VEGF (see Supplementary material online, *Figure S1A*), a significant increase in the ratio of *sFlt1* to *flFlt1* was observed (*Figure 1E*). A similar shift was observed in 3′RNA-seq results, showing an increase in reads at defined poly(A) sites within *Flt1* intron 13 under hypoxic conditions (*Figure 1F*). Whether APA can be specifically targeted to shift FLT1 isoform expression and the functional role of these *Flt1* isoforms in angiogenesis and tissue repair remains unclear.

### 3.2 Introducing selective point mutations in *Flt1^In13pA^* mice activates APA and the expression of soluble Flt1 in the absence of the full-length receptor

Sterically blocking binding of U1 to the *Flt1* exon13/intron13 junction by a complementary morpholino oligonucleotide (MoVE1) dose-dependently induced activation of an alternative polyA site in intron 13 and thus the generation of *sFlt1* in a mouse endothelial cell line (SVEC^17^) *in vitro* (see Supplementary material online, *Figure S1B* and *C*), similarly as in human cells,^23^ and in line with the highly conserved *Flt1* intron 13 nucleotide sequence and C-terminus of the sFLT1 protein between species (see Supplementary material online, *Figure S1D* and *E*). We thus hypothesized that disrupting the binding of U1 to the *Flt1* exon13/intron13 junction could be harnessed to site-specifically interfere with telescripting also *in vivo* and to induce a shift in the balance between *sFlt1* and *flFlt1* expression. Based on this hypothesis, we created a mouse model by mutating the *Flt1* exon13/intron13 5′ splice site by homology-directed repair using CRISPR/Cas9 gene editing with the intent to disrupt the U1 consensus binding sequence (*Figure 2A*). We introduced silent mutations in exon 13 (−4C and −2C) and intron 13 (+2C and +5A) to maintain the FLT1 amino acid sequence during translation.

**Figure 2:**
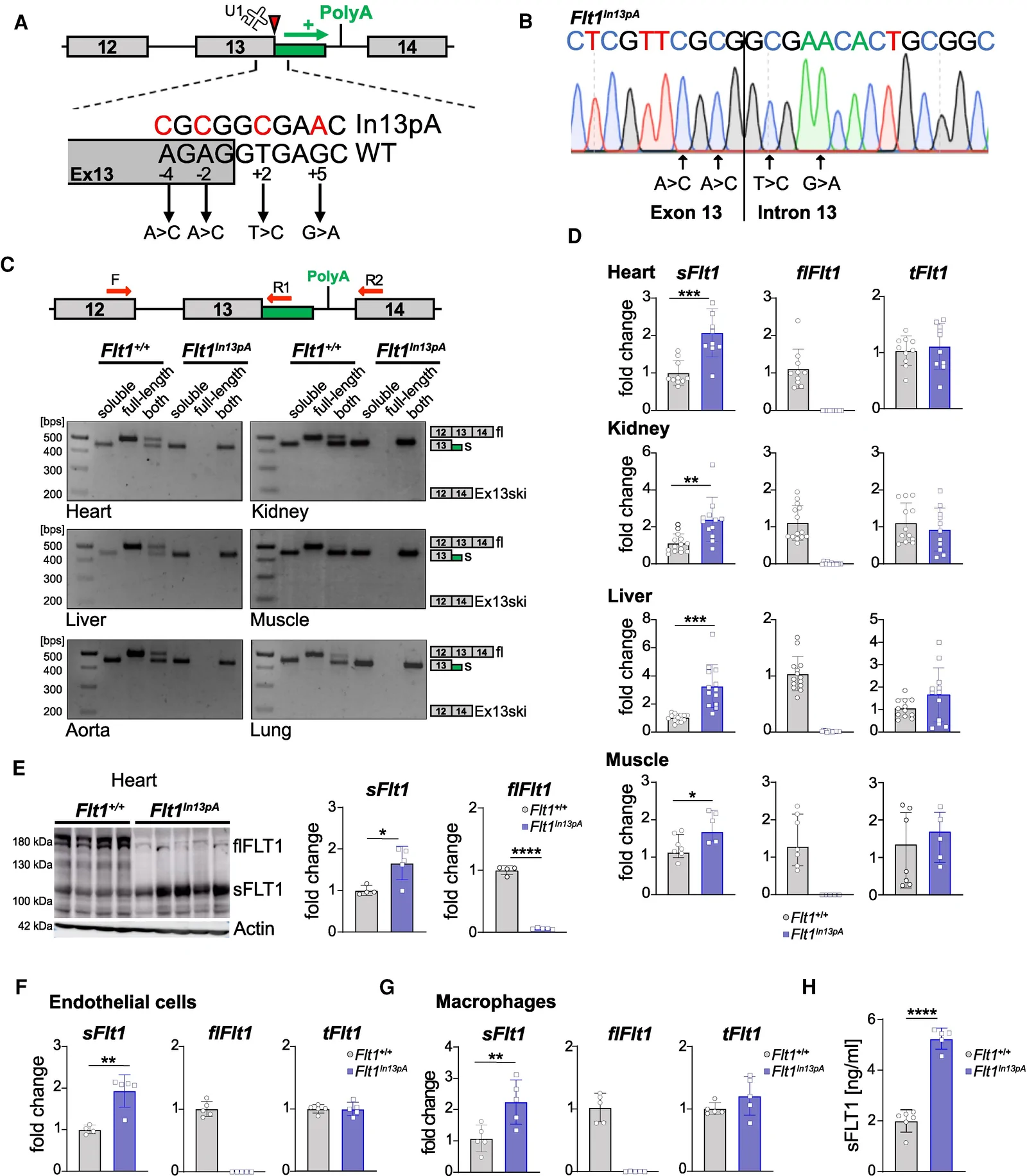
Homozygous *Flt1*^*In13pA*^ mice lack *flFLT1* and display a complete switch towards *sFLT1* expression. (*A*) Introduction of silent genomic mutations in Flt1 exon 13 (−4C and −2C) and in intron 13 (+2C and +5A) by CRISPR/Cas9, (*B*) as confirmed by sequencing of genomic DNA from homozygous *Flt1*^*In13pA/In13pA*^ mice. (*C*) PCR products amplified with primers specific for soluble *Flt1* (F + R1), full length *Flt1* (F + R2), and both *Flt1* isoforms (F + R1 + R2); PCR products using cDNA templates isolated from different organs were analysed by gel electrophoresis. Ex13ski, intron 13 skipping. (*D*) qPCR quantification of *sFlt1*, *flFlt1*, and *tFlt1* transcript expression in different tissues (*n* = 7–14 *Flt1^+/+^* and *n* = 5–13 *Flt1*^*In13pA*^ mice). (*E*) Western blot analysis of heart protein lysate (*n* = 4–5 *Flt1^+/+^* and *n* = 5 *Flt1*^*In13pA*^ mice). (*F–G*) qPCR quantification of sFLT1, flFLT1, and *tFlt1* transcript expression in (*F*) isolated lung endothelial cells (independent endothelial cell isolations from *n* = 4 *Flt1^+/+^* and *n* = 5 *Flt1*^*In13pA*^ mice) and (*G*) bone marrow-derived macrophages (independent BMDM cultures from *n* = 5 mice per genotype). (*H*) sFLT1 levels in cell culture supernatant of untreated *Flt1*^+/+^ and *Flt1*^*In13pA*^ lung endothelial cells measured by ELISA (*n* = 5 *Flt1^+/+^* and *n* = 6 *Flt1*^*In13pA*^ mice). Data represent mean ± SD. Results were analysed by two-tailed, unpaired Student’s *t*-test. *P < 0.05, **P < 0.01, ***P < 0.001, ****P < 0.0001.

F1 founder mice heterozygous for the *Flt1*^*In13pA*^-allele with all four point mutations were genotyped by TaqMan assays (see Supplementary material online, *Figure S2A* and *B*) and subsequently bred to homozygosity (*Figure 2B*). Crossing of heterozygous mice gave rise to offspring close to the expected Mendelian ratio of 1:2:1 (*Flt1*^+/+^: 67, *Flt1*^+/In13pA^: 134, *Flt1*^*In13pA/In13pA*^: 78, Supplementary material online, *Figure S2C*). To analyse if mutating the U1 binding site is sufficient to change specifically the APA-dependent balance of s*Flt1* and fl*Flt1* in *Flt1*^*In13pA/In13pA*^ mice (hereafter referred to as *Flt1*^*In13pA*^ mice), cDNA from the aorta, heart, liver, lung, kidney, and muscle was amplified by non-quantitative three-oligo PCR using two reverse primers specific for the full-length or the truncated soluble isoform, respectively, together with a common forward primer. Polymerase chain reaction products could be detected for both *Flt1* isoforms in all tissue samples in *Flt1*^+/+^ animals. In contrast, *flFlt1* was undetectable in *Flt1*^*In13pA*^ samples, whereas *sFlt1* transcripts were clearly amplified (*Figure 2C*). Steric hindrance of 5′ splice sites has previously been shown to lead to exon skipping or cryptic splice site usage if an alternative intronic polyA signal is absent.^24^ Both alternative splicing events could be excluded since neither an exon 13-skipped product nor any other alternative spliced products were detected using primers flanking exon 13 for amplification (*Figure 2C*).

To precisely characterize the impact of the introduced 5′ splice site mutations on *Flt1* pre-mRNA processing and subsequent protein expression, we performed 3′RACE on RNA isolated from *Flt1*^*In13pA*^ liver tissue. Sequencing of the oligo(dT) primed product revealed a read-through of 115 nucleotides in intron 13, followed by cleavage and polyadenylation (see Supplementary material online, *Figure S2D*). Of these, the first 96 nucleotides encode a unique 32 amino acid sFLT1 tail (including the stop codon).

We analysed tissue samples by real-time RT-qPCR to quantify changes in *Flt1* isoform balance. Tissue from *Flt1*^*In13pA*^ mice showed a significant (approximately twofold) increase in *sFlt1* mRNA expression compared to *Flt1^+/+^* mice, whereas *flFlt1* transcripts were again undetectable. The expression level of total *Flt1* transcripts was unaltered, supporting a complete redirection in isoform expression (*Figure 2D*). Western blot analyses of tissue lysates confirmed the complete loss of transmembrane flFLT1 protein and consequent increase of sFLT1 in *Flt1*^*In13pA*^ heart and liver and corroborated tissue-specific expression of the isoforms closely mirroring the RT-qPCR results (*Figure 2E*, Supplementary material online, *Figure S2E*). Similar gene expression patterns were found in endothelial cells isolated from the lung, as well as in bone marrow-derived macrophages (BMDM) from *Flt1*^*In13pA*^ vs. control animals (*Figure 2F* and *G*). *Flt1*^*In13pA*^ ECs cultivated for 28 h showed a 2.5-fold increase in sFLT1 protein secreted into the culture supernatant compared to the control, as measured by ELISA (*Figure 2H*). These analyses show that genomic mutations blocking U1 snRNP binding to a specific 5′splice site are sufficient to induce a shift in pre-mRNA processing, triggering premature cleavage and polyadenylation, and a complete switch in expression of full-length FLT1 to its endogenous soluble decoy isoform.

### 3.3 *Flt1^In13pA^* mice exhibit normal vascular development

Whereas knockout of *Kdr* leads to embryonic lethality due to a lack of vasculogenesis,^11^ loss of *Flt1* as a negative regulator of angiogenesis results in embryonic death due to an overshooting blood vessel EC growth.^6^ As sFLT1 can still sequester VEGF to limit VEGFR2 signalling,^4,5^ we expected a non-lethal phenotype, with potentially defective vessel formation in our *Flt1*^*In13pA*^ mice that selectively express sFLT1 and lack flFLT1. However, we did not detect any obvious defects during embryonic development, and embryo morphology and crown-rump length were unaltered (*Figure 3A* and *B*). Similarly, postnatal retinal vascular development, evaluated using flat-mounted retinas after fluorescein isothiocyanate (FITC)-conjugated isolectin-B4 staining, was unaltered, with no significant differences in the vascular area, number of branch points, or total vessel length in *Flt1*^*In13pA*^ vs. *Flt1*^+/+^ mice at 5 and 7 days *post partum* (*Figure 3C*). Moreover, the morphology of the heart, liver, and kidney and their weight relative to body weight were unaltered in 8-week-old mice (see Supplementary material online, *Figure S3A* and *B*). No abnormalities or differences in the number of CD31^+^ vessels could be detected in histological sections in these organs of adult *Flt1*^*In13pA*^ mice compared to controls (see Supplementary material online, *Figure S3C* and *D*). Similarly, white blood cell counts, including monocyte levels, remained unchanged (see Supplementary material online, *Figure S3E*). These data provide unprecedented and unequivocal evidence that the presence of sFLT1 in the absence of flFLT1 is sufficient for normal developmental vasculogenesis and pre- and postnatal angiogenesis.

**Figure 3:**
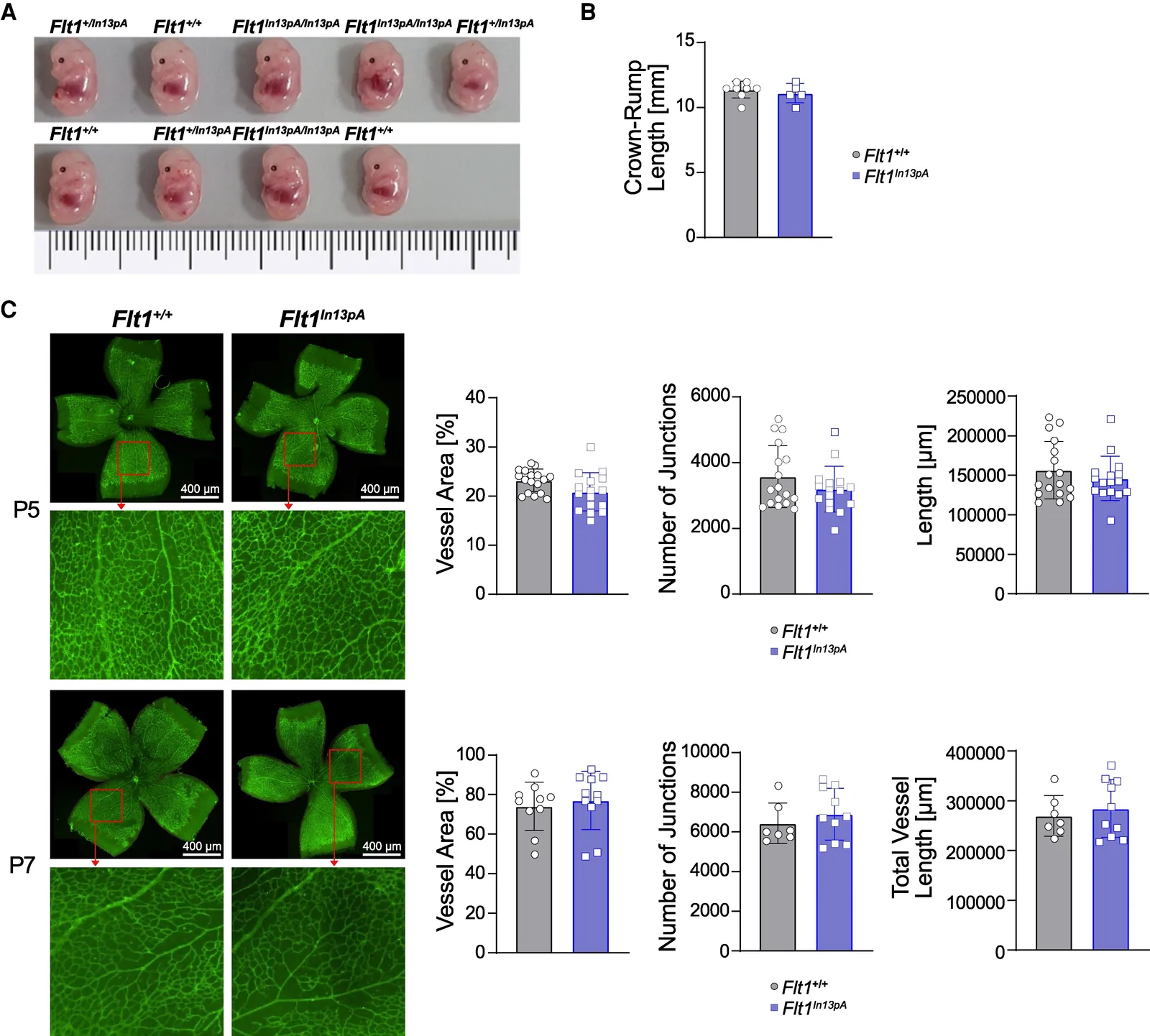
Embryo size and developmental angiogenesis are unaffected in *Flt1*^*In13pA*^ mice. (*A*) Representative photographs of littermate embryos from heterozygous (*Flt1*^+*/In13pA*^) mouse breeding and (*B*) crown-rump length of *Flt1^+/+^* and homozygous *Flt1*^*In13pA*^ embryos at E13.5 (*n* = 8 *Flt1^+/+^* and *n* = 5 *Flt1*^*In13pA*^ mice). (*C*) Analysis of vessel area, junction numbers, and total vessel length of isolectin-B4-stained retinal blood vessels at Day 5 (P5, *n* = 16 *Flt1^+/+^* and *n* = 17 *Flt1*^*In13pA*^ mice) and Day 7 (P7, *n* = 7–10 *Flt1^+/+^* and *n* = 10–11 *Flt1*^*In13pA*^ mice). Data represent mean ± SD. Results were analysed by two-tailed, unpaired Student’s *t*-test.

### 3.4 Redirection of flFLT1 towards sFLT1 isoform expression unleashes angiogenesis after injury and improves cardiac remodelling after MI

Different from vessel formation during development, the postnatal vascular system adapts to pathological cues by angiogenesis to maintain tissue homeostasis, i.e. by sprouting of capillaries from the pre-existing vasculature, e.g. in ischaemic tissue.^25^ Given the high expression of FLT1 in the heart as well as the shift in expression towards *sFlt1* in the infarcted myocardium, we further scrutinized the angiogenic response after ischaemic injury in mice subjected to permanent MI. Left ventricular function, assessed by echocardiography, was similar between groups at baseline. After MI, *Flt1*^*In13pA*^ mice were protected from the pronounced systolic dysfunction observed in *Flt1*^+/+^ mice, as evident from a stronger EF, FS, and a smaller left ventricular inner diameter in systole (LVID) (*Figure 4A*). Heart rates were comparable (see Supplementary material online, *Figure S4A*). Kaplan–Meier analysis indicated a trend towards improved survival over 28 days post-MI (*Figure 4B*). No differences in infarct size were observed between genotypes (*Figure 4C*). Notably, the capillary density in the infarct border zone was higher in *Flt1*^*In13pA*^ mice (*Figure 4D*), but not in the remote area (see Supplementary material online, *Figure S4B*). We also observed a decrease in relative heart weight and cardiomyocyte size (*Figure 4E* and *F*) and less fibrosis in the infarct border zone (*Figure 4G*). Flow cytometric analysis of the cardiac inflammatory cell infiltrate at day 3 after MI furthermore demonstrated a reduction in monocyte accumulation and a trend towards reduced counts of recruited monocyte-derived inflammatory TIM4^−^ MHCII^+^ and TIM4^−^ MHCII^−^ macrophages but increased TIM4^+^ resident macrophages, using gating strategies previously established;^26^ neutrophil levels were unaltered (see Supplementary material online, *Figure S4C–E*). Lower cardiac monocyte accumulation was paralleled by a reduction in monocyte numbers in the blood and bone marrow (see Supplementary material online, *Figure S4F* and *G*). These data indicate that a shift in the expression from flFLT1 to sFLT1 accelerates angiogenesis, reduces monocyte recruitment, and promotes tissue repair after ischaemic injury.

**Figure 4:**
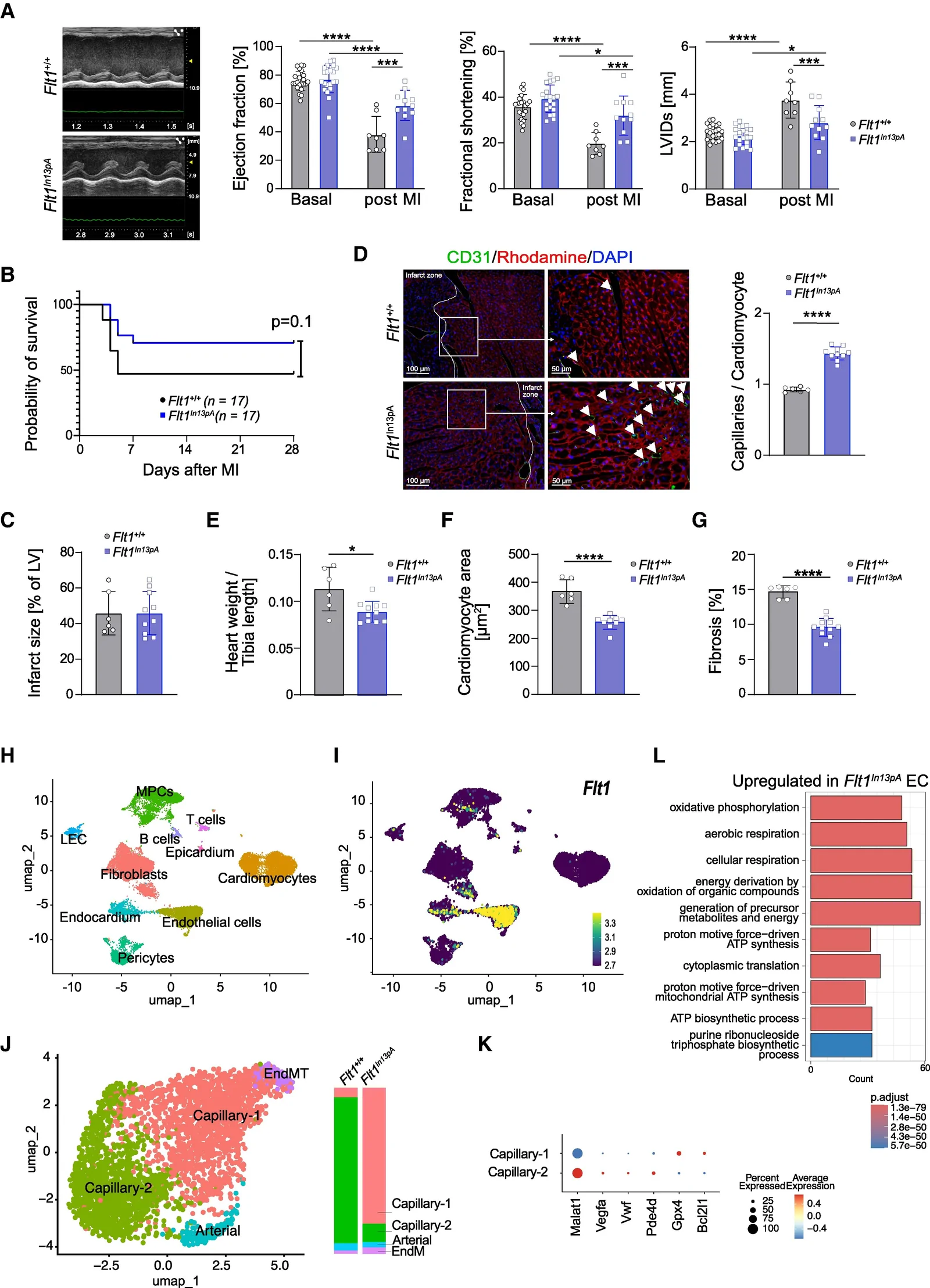
Redirection of flFLT1 expression towards sFLT1 promotes postischaemic angiogenesis and improves cardiac function after myocardial infarction. *Flt1*^+/+^ and *Flt1*^*In13pA*^ mice were subjected to MI injury. (*A*) M-Mode echocardiogram and quantification of ejection fraction (%), fractional shortening (%), and internal left ventricular end-systolic diameter (mm) under basal conditions (*n* = 22 *Flt1*^+/+^ and *n* = 21 *Flt1*^*In13pA*^ mice) and at Day 28 (*n* = 8 *Flt1*^+/+^ and *n* = 11 *Flt1*^*In13pA*^ mice) after MI. Data were analysed by one-way ANOVA followed by Tukey’s *post hoc* test. (*B*) Kaplan–Meier survival analysis over 28 days post-MI (*n* = 17 mice per genotype). (*C*) Quantification of the relative infarct size at 28 days post-MI (*n* = 6 *Flt1*^+/+^ and *n* = 10 *Flt1*^*In13pA*^ mice). Results were analysed by two-tailed, unpaired Student’s *t*-test. (*D*) Representative immunofluorescence staining of CD31^+^ capillaries and Rhodamine staining to demarcate cardiomyocytes in the infarcted myocardium of *Flt1*^+/+^ (*n* = 6) and *Flt1*^*In13pA*^ (*n* = 10) mice at 28 days post-MI (scale bars, 50 μm). Quantification of CD31^+^ capillaries relative to the number of cardiomyocytes. (*E*) Relative heart weight, (*F*) cardiomyocyte area, and (*G*) border zone fibrosis in the infarcted myocardium of *Flt1*^+/+^ (*n* = 6) and *Flt1*^*In13pA*^ (*n* = 10) mice at 28 days post-MI. Results were analysed by unpaired Student’s *t*-test. Data represent mean ± SD. (*H–K*) Single-nuclei analysis of the left ventricle on Day 28 after myocardial infarction. (*H*) UMAP of major cardiac cell types in total cells from *Flt1*^+/+^ and *Flt1*^*In13pA*^ hearts. (*I*) Feature plot showing *Flt1* expression across different clusters. (*J*) UMAP of subclustered endothelial cells with their relative proportions among genotypes presented as stacked bar graphs. (*K*) Dotplots showing marker genes for different capillary EC and cardiomyocyte states. (*L*) GO-term analysis upregulated in *Flt1*^*In13pA*^ ECs. *P < 0.05, ***P < 0.001, ****P < 0.0001.

To further evaluate cardiac cell states, snRNA-seq was performed on ventricular tissue at 28 days after MI. Unsupervised clustering identified cardiomyocytes (*Tnnt2*, *Ryr2*), fibroblasts (*Dcn*, *Pdgfra*, *Col1a1*), EC (*Cdh5*, *Pecam1*, *Kdr*), endocardial cells (*Cdh5*, *Pecam1*, *Npr3*), lymphatic EC (*Flt4*, *Lyve1*, *Prox*), pericytes (*Notch3*, *Pdgfrb*), cells of the mononuclear phagocyte system (MPCs) (*Lyz2, Adgre1*), T (*Cd3e*) and B (*Cd19*) lymphocytes, as well as epicardial cells (*Wt1*) (*Figure 4H*, and Supplementary material online, *Figure S5A* and *B*). *Flt1* expression was mainly confined to EC but could also be detected in some endocardial cells, fibroblasts, and macrophages (*Figure 4I*). Selecting ECs for reclustering, we could discriminate arterial EC (*Gja*), cells undergoing endothelial–mesenchymal transformation (EndMT, characterized by *Acta2* and decreased EC marker expression), and two capillary EC clusters (*Emcn*) (*Figure 4J*, Supplementary material online, *Figure S5C* and *D*). GO enrichment of differentially expressed genes (DEGs) between endothelial subclusters highlighted apoptotic regulation and mitochondrial/organelle membrane functions in Capillary-1 EC, including the anti-apoptotic genes *Gpx4* and *Bcl2l1*. In Capillary-2 ECs, DEGs were enriched for IκB phosphorylation (consistent with NF-κB activation), cadherin-mediated cell–cell adhesion, and cell proliferation, with higher expression of hypoxia- and inflammation-associated genes *Vegfa*, *Malat1*, and *Pde4d*^27^ (see Supplementary material online, *Figure S6A*, *Figure 4K*). These data suggest that Capillary-1 ECs are more quiescent and homeostatic than Capillary-2 ECs, which are activated, proliferate more, and express an inflammatory phenotype. A significant increase in the frequency of Capillary-1 ECs and a reduction of Capillary-2 ECs were observed in the *Flt1*^*In13pA*^ mice (*Figure 4J*). Interestingly, GO terms upregulated across ECs and cardiomyocytes in *Flt1*^*In13pA*^ compared to *Flt1*^+/+^ mice included metabolic pathways such as oxidative phosphorylation and aerobic respiration, suggesting greater energy efficiency (*Figure 4L*, Supplementary material online, *Figure S6D*). Analysis of the phenotype of fibroblasts revealed a reduction in fibroblast matrisome scores^28^ in the *Flt1^In13pA^* hearts (see Supplementary material online, *Figure S7A*), suggesting attenuated myofibroblast activation and decreased ECM production. Collectively, these data indicate that the *Flt1^In13pA^* allele provides a cardioprotective phenotype, including reduction of cardiac stress, fibrosis, and maladaptive remodelling, plus an improved LV ejection fraction.

It has previously been shown that FLT1 binds PlGF for its removal from the circulation,^29^ an important cytokine contributing to adaptive cardiac remodelling during chronic pressure overload.^30^ We therefore evaluated whether the absence of membrane-bound FLT1 in *Flt1*^*In13pA*^ mice entails an accumulation of PlGF. We indeed noted elevated PlGF levels in *Flt1^In13pA^* mice at baseline and after MI compared to *Flt1*^+/+^ mice (see Supplementary material online, *Figure S7B*).

Next, we induced hindlimb ischaemia by femoral artery ligation to confirm that a shift in FLT1 isoform expression regulates angiogenesis in a second *in vivo* model of ischaemic tissue injury *in vivo*.^16^ Indeed, an increased CD31^+^ capillary density relative to the number of myocytes was evidenced at day 14 after injury in ischaemic but not the non-ligated (see Supplementary material online, *Figure S8A* and *B*) gastrocnemius muscles of *Flt1*^*In13pA*^ vs. *Flt1*^+/+^ mice. We also noted a reduction in macrophage accumulation in the injured muscles in *Flt1*^*In13pA*^ mice after injury (see Supplementary material online, *Figure S8C*).

### 3.5 flFLT1 constrains VEGFR2 signalling and angiogenesis

To further mechanistically dissect the impact of the ratio between soluble and full-length FLT1 on angiogenesis, we used primary ECs isolated from *Flt1*^*In13pA*^ and *Flt1*^+/+^ mice (see Supplementary material online, *Figure S9A*) in a tube formation assay. In line with the enhanced angiogenic response observed *in vivo*, more tubes formed in *Flt1*^*In13pA*^ than in *Flt1*^+/+^ EC cultures (*Figure 5A*). Cell proliferation was increased in *Flt1*^*In13pA*^ compared to *Flt1*^+/+^ ECs (see Supplementary material online, *Figure S9B*), and Western blot analyses showed an increased VEGFR2 phosphorylation in *Flt1*^*In13pA*^ compared to *Flt1*^+/+^ ECs, without differences in *Kdr* mRNA (not shown) or total VEGFR2 protein levels (*Figure 5B*). To explore differences in the transcriptional signatures of *Flt1*^+/+^ and *Flt1*^*In13pA*^ ECs under hypoxic conditions, we performed bulk RNA sequencing and pathway enrichment analysis of DEGs using GSEA. This unveiled a significant enrichment in DEGs involved in angiogenesis (*Figure 5C*, Supplementary material online, *Figure S9C*) and the upregulation of genes controlling angiogenesis, including C-X-C chemokine receptor type 4 (*Cxcr4*), nitric oxide synthase 3 (*Nos3*), delta-like 4 (*Dll4*), angiopoietin 2 (*Angpt2*), and *Flt4*^31^ in *Flt1*^*In13pA*^ vs. *Flt1*^+/+^ ECs (*Figure 5D*).

**Figure 5:**
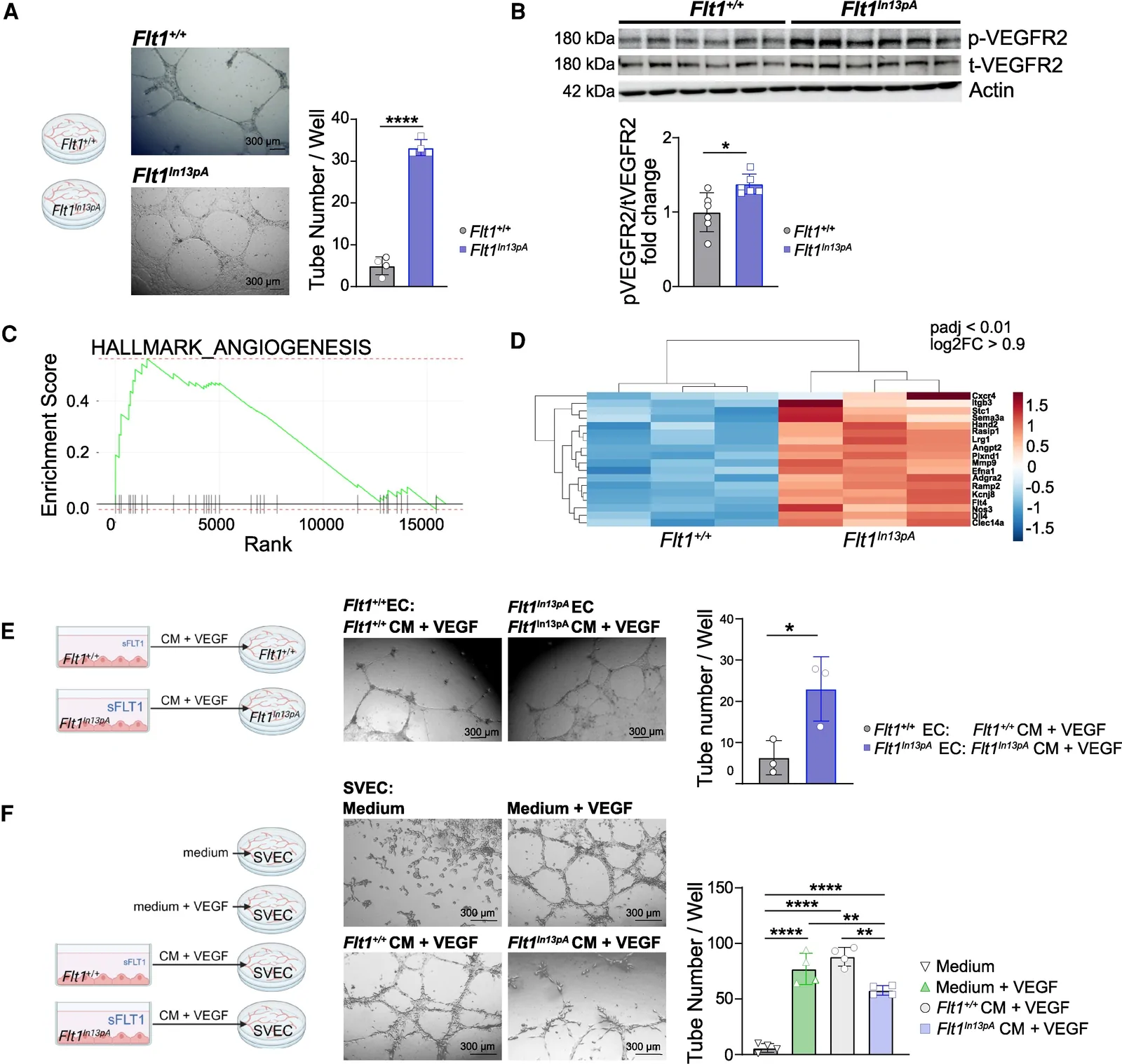
flFLT1 controls the angiogenic response in endothelial cells. (*A*) Tube formation assay of *Flt1^+/+^* and *Flt1*^*In13pA*^ mouse lung endothelial cells cultured for 24h (independent endothelial cell isolations from *n* = 4 mice per genotype). Representative phase contrast micrographs (scale bars, 300 μm) and quantification of tubes. Results were analysed by two-tailed, unpaired Student’s *t*-test. (*B*) Phosphorylated VEGFR2 (p-VEGFR2) and total VEGFR2 (t-VEGFR2) were analysed in untreated *Flt1^+/+^* and *Flt1*^*In13pA*^ mouse lung endothelial cell lysates by Western blotting (*n* = 6 per genotype). Results were analysed by two-tailed, unpaired Student’s *t*-test. (*C, D*) Bulk RNA sequencing of *Flt1*^+/+^ and *Flt1*^*In13pA*^ lung endothelial cells exposed to hypoxia over 24 h (*n* = 3 experiments performed with pooled endothelial cells isolated from *n* = 8 mice). (*C*) Gene set enrichment analysis (GSEA) of the angiogenesis gene set against MSigDB hallmark gene sets with a significant cutoff determined by padj < 0.05 plot. (*D*) Heatmap of RNA-seq data for angiogenesis-associated genes. (*E*) Tube formation assay of *Flt1^+/+^* and *Flt1*^*In13pA*^ mouse lung endothelial cells incubated with VEGF-supplemented conditioned medium (CM) of *Flt1^+/+^* or *Flt1*^*In13pA*^ lung endothelial cells (independent endothelial cell isolations from *n* = 3 mice per genotype). Results were analysed by two-tailed, unpaired Student’s *t*-test. Representative phase contrast micrographs and quantification of tubes. (*F*) Tube formation assay of SVEC incubated with unconditioned media, VEGF-supplemented unconditioned media, or VEGF-supplemented conditioned medium (CM) of *Flt1^+/+^* or *Flt1^In13pA^* lung endothelial cells (*n* = 4 per condition). Representative phase contrast micrographs (scale bar, 300 μm) and quantification of tubes. Results were analysed using one-way ANOVA followed by Tukey’s *post hoc* test. Data represent mean ± SD. *P < 0.05, **P < 0.01, ***P < 0.001, ****P < 0.0001.

These findings were independent of the increased levels of sFLT1 in the supernatants from *Flt1*^*In13pA*^ compared to *Flt1*^+/+^ EC cultures (*Figure 2H*), which contradicts the previous finding that adding sFLT1 to EC culture medium suppresses tube formation.^32^ To further address this issue, we analysed the VEGF-dependent angiogenic effects in *Flt1*^+/+^ ECs grown in *Flt1*^+/+^ EC-conditioned medium (containing sFLT1 at a concentration of 2000 pg/mL) and *Flt1*^*In13pA*^ ECs in *Flt1*^*In13pA*^ EC-conditioned medium (with sFLT1 at 5242 pg/mL). In response to exogenous VEGF, *Flt1*^*In13pA*^ cells again formed more tubes than *Flt1*^+/+^ cells, despite the increased sFLT1 concentration (*Figure 5E*). In contrast, when investigating VEGF-dependent angiogenic effect in a lymphoid endothelial cell line (SVEC cells^17^) grown in supernatant from *Flt1*^+/+^ or *Flt1*^*In13pA*^ cells, we observed the expected inhibition of tube formation in the presence of *Flt1*^*In13pA*^ compared to *Flt1*^+/+^ EC-conditioned medium (*Figure 5F*). These data collectively indicate that *Flt1*^*In13pA*^ ECs have a higher intrinsic angiogenic potential, presumably linked to the absence of suppressive flFLT1 signalling, despite an increased expression of sFLT1.

VEGF-B and PlGF selectively bind FLT1 and can thus indirectly amplify VEGF-VEGFR2 responses by displacing VEGF from FLT1. PlGF, however, also has weak pro-angiogenic activity.^33^ To test the effects of these ligands in our model, we stimulated ECs with VEGF, VEGF-B, PlGF, or their combination. As expected, VEGF induced strong proliferation of *Flt1*^+/+^ ECs, and this response was even further enhanced in *Flt1*^*In13pA*^ ECs (see Supplementary material online, *Figure S9D*). VEGF-B did not affect EC proliferation in both *Flt1*^+/+^ and *Flt1*^*In13pA*^ cultures. PlGF increased cell proliferation significantly in *Flt1*^+/+^ but not in *Flt1*^*In13pA*^ ECs, in line with its pro-angiogenic activity via FLT1.^33^ The combination of all three ligands significantly increased EC proliferation in *Flt1*^+/+^ ECs and even further in *Flt1*^*In13pA*^ ECs (see Supplementary material online, *Figure S9D*). We also assessed VEGFR2 phosphorylation after 5 min of exposure to these ligands by Western blotting (see Supplementary material online, *Figure S9E*). The phosphorylation patterns of VEGFR2 largely followed EC proliferative responses.

### 3.6 flFLT1 controls VEGF-induced chemotactic responses in macrophages *in vitro*

Monocytes lack VEGFR2 expression, and FLT1 has previously been shown to mediate their VEGF-induced chemotactic responses.^34^ We therefore tested whether the FLT1 isoform switch affects BMDM migration. While we noted equal migratory responses of *Flt1*^*In13pA*^ and *Flt1*^+/+^ macrophages towards CCL2 and FCS after 3.5 h of migration (data not shown), VEGF elicited no chemotactic activity in *Flt1*^*In13pA*^ macrophages compared to efficient responses in *Flt1*^+/+^ macrophages (see Supplementary material online, *Figure S10A*). Similarly, the VEGF-induced *in vivo* accumulation of monocytes and macrophages in an air pouch model was inhibited in *Flt1*^*In13pA*^ compared to *Flt1*^+/+^ mice (see Supplementary material online, *Figure S10B*). These data indicate that increased expression of sFLT1 and the absence of flFLT1 in *Flt1*^*In13pA*^ macrophages impair their migration towards VEGF. Furthermore, these findings align with the reduced recruitment of macrophages to the infarcted heart observed in *Flt1*^*In13pA*^ mice.

## 4. Discussion

Using FLT1 as a model gene, we here for the first time selectively activated its alternative intronic polyA signal in intron 13 by introducing silent mutations at the upstream 5′ splice site to completely redirect gene isoform expression from flFLT1 towards the sFLT1 isoform. Alternative polyadenylation is a widespread regulatory mechanism that can generate shortened mRNAs and subsequently truncated protein isoforms. Although increasingly regarded as a crucial regulatory mechanism in many diseases, it has been challenging to modulate the generation of specific gene isoforms to address their functional role. Importantly, sFLT1, dominantly produced in the generated *Flt1*^*In13pA*^ mice, is identical to the endogenously produced isoform found *in vivo* and increased in different pathologies, including heart failure.^13,35,36^

Different genetically modified mouse models have been generated, including the conditional Cre-*lox*P-mediated full-body knockout of FLT1 in neonatal and adult mice^37^ and the deletion of a FLT1 kinase domain resulting in the expression of inactive membrane-bound flFLT1 and sFLT1,^10^ which show vascular normalcy at steady state. Although widely accepted to function as a decoy receptor to sequester excess VEGF without effective signal transduction in the endothelium,^4–6^ it has, however, previously been difficult to dissect the contribution of sFLT1 and flFLT1 to these processes. The complete endogenous shift towards sFLT1 with concomitant knockout of *flFlt1* in our mouse model allowed us to uncover that the two isoforms of FLT1 affected developmental and postnatal reparative angiogenesis differentially in adult mice. We could show that the expression of sFLT1, in the absence of flFLT1 in *Flt1*^*In13pA*^ mice, was sufficient to allow the development of a normal vasculature both during embryogenesis and postnatally. *Flt1*^*In13pA*^ mice were viable and fertile and displayed no abnormalities in organ size or vascularization. These findings are in line with previous findings obtained *in vitro*, demonstrating that the expression of *sFlt1* alone is sufficient to rescue branching morphogenesis in murine *Flt1*^-/-^ embryonic stem cell-derived vessels.^38,39^

In contrast, tube formation and regulation of genes promoting angiogenic cell responses in endothelial cells *in vitro*, and angiogenesis in response to ischaemia in mice, were differentially regulated by the two FLT1 isoforms, pointing towards a cell-intrinsic dominant negative role of flFLT1 that restrains reparative angiogenesis in adult mice. A similar mechanism may operate in *Flt1* tyrosine kinase-deficient (*Flt1 tk*^-/-^) mice,^10^ where non-signalling FLT1 can still serve as a local VEGF sink and form non-signalling FLT1 and FLT1/VEGFR2 receptor dimers to inhibit angiogenic responses in ECs. *Flt1 tk*^-/-^ mice display a reduced microvascular density after femoral artery ligation, an effect thought to stem from the concomitant defect in pro-angiogenic macrophage accumulation.^40^ In *Flt1*^*In13pA*^ mice, the shift in expression from flLFT1 to sFLT1 enhanced angiogenesis in the skeletal and cardiac muscle in response to ischaemic injury, despite reduced macrophage accumulation in the injured tissues of *Flt1*^*In13pA*^ mice. This suggests that lifting the flFLT1-mediated brake on VEGFR2 angiogenic signalling in ECs triggered by locally increased VEGF bioavailability is sufficient to promote neovascularization. Such a shift towards reduced *flFlt1* expression in hypoxic ECs *in vitro* may extend the concept of a pro-inflammatory environment triggering vessel sprouting in wound angiogenesis^41^ by modulating cellular FLT1 availability. Lowered cellular FLT1 expression may serve as a trigger to increase EC responsiveness to angiogenic stimuli during ischaemia, even when serum sFLT1 levels are elevated, as seen in mice and patients^13^ after MI, and possibly also derived from its proteolytic release.

Other VEGF family ligands, such as VEGF-B and PlGF, may also increase in response to ischaemic injury. Endothelial cell proliferation and VEGFR-2 phosphorylation were augmented in *Flt1*^*In13pA*^ compared with *Flt1*^+/+^ ECs also in response to the combination of VEGF, VEGF-B, and PlGF.

Monocytes/macrophages that accumulate in the infarct border region promote post-MI angiogenesis.^42^ In *Flt1*^*In13pA*^ mice, the absence of flFLT1 likely prevents monocyte/macrophage sensing of the VEGF gradient.^34^ This likely also impaired the VEGF-induced chemotactic responses of *Flt1*^*In13pA*^ macrophages *in vitro* and in the air pouch model *in vivo*. The VEGF-driven chemotactic responses in macrophages furthermore imply cell-type-specific differences in FLT1 responses, in contrast to the weak FLT1 signalling responses in ECs.^10^ We furthermore identified an important role of FLT1 in the control of monocyte levels in the blood and bone marrow after MI. This is in line with the lower overall inflammation as a result of the diminished monocyte accumulation and reduced production of inflammatory mediators, such as IL-1β, known to trigger myelopoiesis after MI.^43^ FLT1 has previously been reported to promote hematopoietic stem cell mobilization after myelosuppression,^44^ a mechanism that also replenishes monocyte levels in the bone marrow after MI. Of note, the diminished monocyte/macrophages and sFLT1 produced by them may also contribute to the locally increased VEGF bioavailability and consequent neovascularization of the ischaemic tissue in the *Flt1*^*In13pA*^ mice. However, the full scope of sFLT1 functions remains unclear. For example, sFLT1 can directly bind to macrophages and regulate their inflammatory functions.^45^ sFLT1 has also been reported to mitigate the expression of pro-inflammatory cytokines in disease models such as atherosclerosis, cardiac hypertrophy, and dysfunction.^46,47^

Previous studies have shown that improved neovessel formation after MI mitigates scarring and worsening of heart function and that VEGF dosage critically controls cardiac remodelling after MI.^48,49^ In line with this, we noted reduced cardiac dysfunction after MI in the *Flt1*^*In13pA*^ mice. These data align and are consistent with recent observations in zebrafish, in which *Flt1*^-/-^ mutant hearts display enhanced coronary revascularization and improved cardiac regeneration following cryoinjury.^50^ Also, cardiomyocyte overexpression of VEGF-B, which binds FLT1 and indirectly enhances VEGF-A/VEGFR2 signalling and coronary endothelial growth, prevents the decline in cardiac function after MI.^51^ Single-nucleus RNA-seq of hearts at 28 days after MI revealed that the improved cardiac function in *Flt1*^*In13pA*^ mice was associated with a shift in the transcriptional profile of capillary ECs from genes associated with hypoxia and environmental responses to genes characteristic of a more quiescent EC state. Furthermore, based on transcriptome analysis, cardiomyocytes in the *Flt1*^*In13pA*^ mice appeared to acquire a more mature phenotype than in the control mice, which displayed a stressed and hypoxic phenotype. Fibroblasts showed a reduced matrisome score,^28^ suggesting attenuated myofibroblast activation and ECM production. These findings corroborate the conclusion that the improved endothelial recovery after MI in the *Flt1*^*In13pA*^ mice accelerates cardiac repair and attenuates the inflammatory cardiac microenvironment. The reduction in post-MI fibrosis may reflect lower maladaptive activation of cardiac fibroblasts as a consequence of improved vascularization and lower (macrophage-driven) inflammatory signalling in the heart.^52–54^ Notably, the functional improvement despite unchanged infarct size supports a setting in which vascular recovery causes favourable cardiac remodelling, leading to improved contractile efficiency of the viable myocardium. Such compensatory mechanisms have been well described following MI and delay heart failure development, even though they can eventually transition to progressive remodelling and ultimately can lead to systolic dysfunction.^55^ However, several limitations need to be acknowledged: First, EF is a global volumetric parameter that does not adequately capture regional wall–motion abnormalities when a compensatory hyperkinesis of nonaffected myocardium is present. Second, our follow-up of cardiac function was limited to the 4-week time point after MI, which may not fully reflect long-term remodelling and potential functional decline. Future studies need to determine the durability of the protective phenotype in *Flt1*^*ln13pA*^ mice. Furthermore, the analysis should include more sensitive measures of myocardial mechanics, such as global longitudinal strain, to detect subclinical myocardial dysfunction not captured by conventional EF measurements.

FLT1 contributes to the clearance of PlGF from the circulation.^29^ Clinical measurements of sFLT1 that inversely correlate with levels of PlGF in serum during preeclampsia have suggested the removal of free PlGF by sFLT1.^56^ Our data showing an accumulation of PlGF in *Flt1^In13pA^* mice provide evidence that flFLT1 plays an important role in the clearance of PlGF *in vivo*. PlGF signals through FLT1^8^ to promote angiogenesis and to act on myeloid cells, activities that would be absent in *Flt1*^*ln13pA*^ mice. However, PlGF can also signal via Neuropilin (NRP1) independently of VEGFR1.^57^ In the heart, the PlGF-NRP1 axis was shown to expand the subset of NRP1^+^ resident macrophages in response to cardiac pressure overload, thereby enabling protective adaptive hypertrophic remodelling of cardiomyocytes.^30^ Increased PlGF levels in our mouse model may thus have contributed to the trend towards expansion of resident macrophages in response to MI and to the improved cardiac function in *Flt1*^*In13pA*^ mice.

Despite several attempts, therapeutic enhancement of angiogenesis to improve cardiac healing has not yet succeeded. Although increased VEGF provides strong angiogenic signalling, it also promotes vascular leakage and leukocyte recruitment, precluding its therapeutic use in the treatment of myocardial ischaemia.^58^ Mechanistically, the net effect of our *Flt1*^*In13pA*^ approach is somewhat similar to the effect of the previously published AAV-VEGF-B overexpression that inhibits VEGF-A binding to FLT1, shifting its activity towards angiogenic VEGFR-2.^59^ However, VEGF-B function is influenced by its cellular source,^60^ and its ability to promote cardiac hypertrophy may increase the risk of arrhythmias^61^ and adipose metabolism.^62–64^ Here, we show that the local balance of flFLT1 vs. sFLT1 expression represents a critical regulator of angiogenesis. Our approach to shift the local balance between flFLT1 and sFLT1 provides a novel therapeutic approach to improve angiogenesis, beneficial for tissue repair after MI,^48^ by sensitizing endothelial cells to VEGF signalling without the requirement for detrimental increases in its concentration, and to constrain inflammation.

Considering eventual therapeutic use, morpholino phosphorodiamidate oligonucleotides (AMOs) have been shown to block U1 binding to the *Flt1* 5′ splice site in intron 13 to generate sFLT1 in cultured cells.^23^ This could be applied via local delivery to the injured tissue by nanobodies or other targeting strategies.^65^ Intriguingly, by this mechanism, the endogenous gene expression is redirected, without the risks of unselective overexpression of angiogenic mediators or off-target effects in cells that do not express the target.^66^ This would represent an advantage over the possible therapies based on VEGF-A and VEGF-B that could have potentially harmful side effects, such as increased vascular permeability, tissue inflammation, or cardiac hypertrophy.

In a broader context, and given the widespread abundance of APA signals,^67^ our approach to target telescripting at the 5′ splice site to induce APA may serve as a blueprint for further exploration of the function of truncated transcripts with alternative 3′UTRs. Our study also implies that often overlooked mutations affecting U1 binding to the 5′ splice site could profoundly alter protein functionality, which should be considered in future genetic studies.

Overall, our findings demonstrate that harnessing APA could be used to explore the endogenous function of many still-uncharacterized truncated transcript isoforms. Here, we exemplify this by our exciting new finding showing that shifting the expression from flFLT1 towards its soluble isoform improves ischaemia-driven neoangiogenesis and cardiac healing and inhibits inflammatory macrophage accumulation while leaving normal developmental vasculogenesis unaltered.

Translational perspective We here demonstrate that harnessing APA can be used to explore the endogenous function of truncated transcript isoforms. As one example, we show that shifting the expression from flFLT1 towards its soluble isoform improves ischaemia-driven neoangiogenesis and cardiac healing while leaving normal developmental vasculogenesis unaltered. Given the widespread abundance of alternative polyadenylation signals, we anticipate our findings as a starting point and methodological blueprint for further exploring the function of many still-uncharacterized truncated protein isoforms generated by APA, opening unprecedented opportunities for future basic research and clinical therapies.

## Supplementary Material

cvag198_Supplementary_Data

## Data Availability

The data that support the findings of this study are available from the corresponding author upon request. Sequencing data have been deposited in the BioStudies database, with accession numbers S-BSST2768 (bulk RNAseq of ECs) and S-BSST2762 (snRNAseq of *Flt1*^+/+^ and *Flt1*^*In13pA*^ hearts).
