# Supplementary material for: Redirecting full-length FLT1 expression towards its soluble isoform by targeting alternative polyadenylation promotes postischaemic angiogenesis
Source: Cardiovasc Res. 2026 Oct 5;122(15):2160–76. doi: 10.1093/cvr/cvag198 (PMC13742059; doi:10.1093/cvr/cvag198)
Supplement: Supplementary file 1 [file cvag198_supplementary_data.zip › CVR-2025-1778_R2_Supplemental Figures.pdf]

Supplemental Figure 1

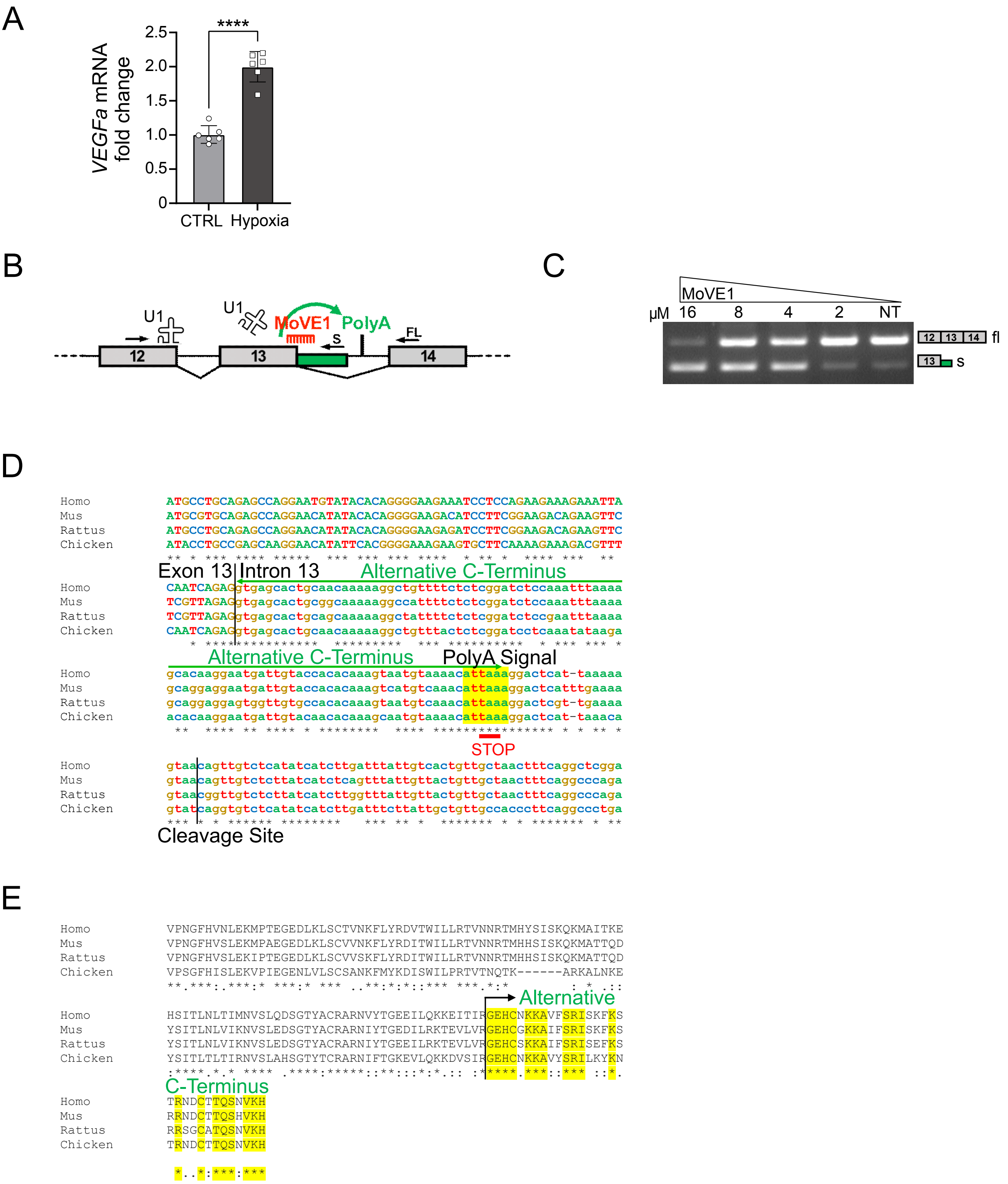

Supplemental Figure 1. (**A**) qPCR analysis of *Vegfa* expression in lung endothelial cells exposed to hypoxia over 24 h (n=6 experiments performed with pooled endothelial cells isolated from n=8 mice). Results were analyzed by two-tailed, unpaired Student's t-test, ****p<0.0001.(**B**) Schematic representation of morpholino oligonucleotide (MoVE1) binding to *Flt1* intron 13/exon13 5′-splice site, thereby blocking binding of U1 snRNP resulting in activation of an alternative cleavage-polyadenylation (polyA) signal within intron 13. (**C**) Dose-dependent treatment of mouse SVEC with MoVE1 results in increased *sFlt1* and concomitant decreased *flFlt1* transcript expression compared to non-treated (NT) cells. (**D**) Interspecies conservation of *Flt1* intron 13 nucleotide sequence and (**D**) of C terminal sFLT1 protein.

Supplemental Figure 2

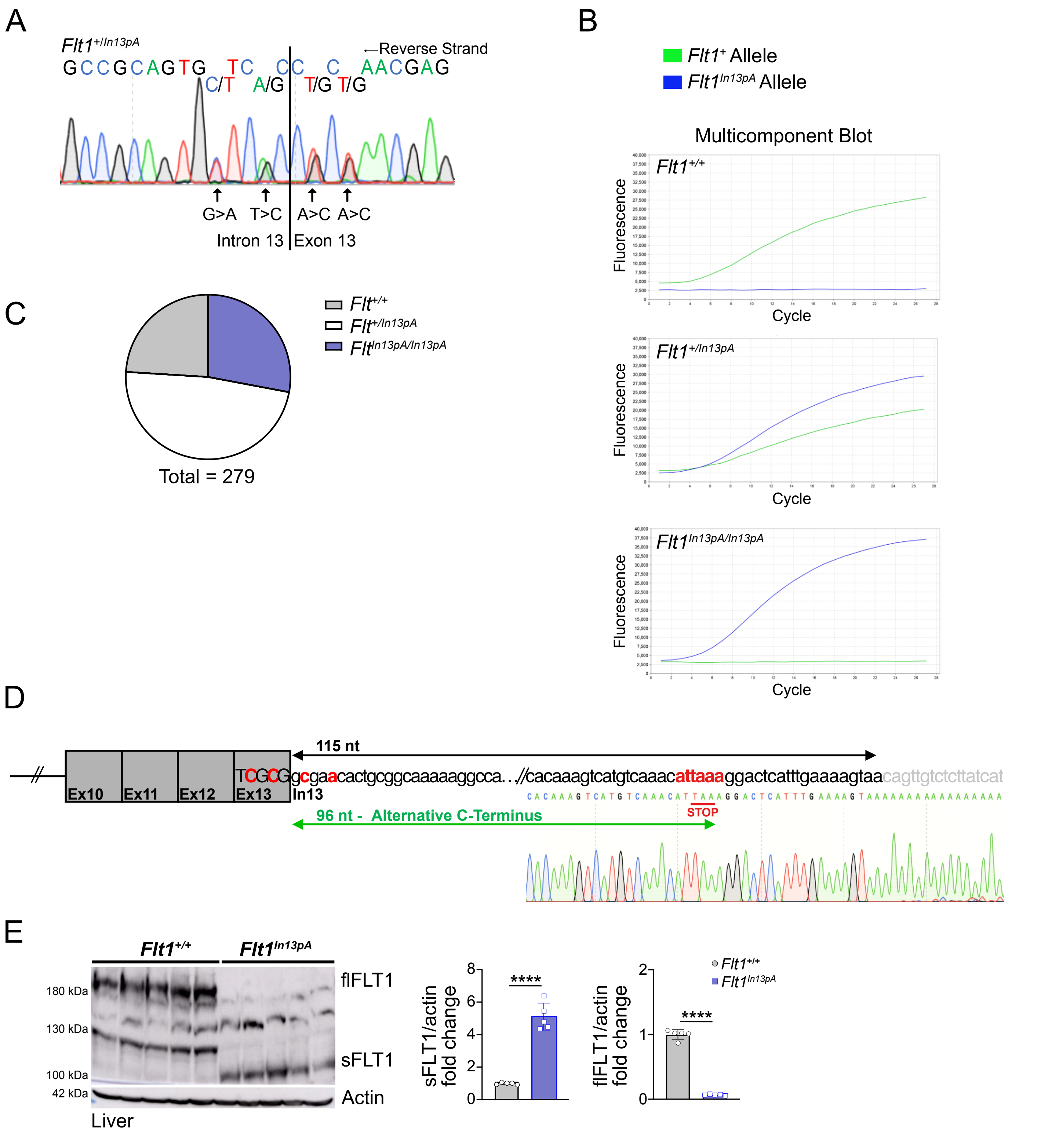

Supplemental Figure 2. (**A**) Genomic DNA sequencing chromatogram of heterozygous *Flt1*$^{+/In13pA}$ F1 founder mice. (**B**) TaqMan multicomponent plot obtained from genotyping *Flt1*$^{+/In13p}$ mice carrying one *Flt1*$^{+}$ and *Flt1*^{In13p} allele. (**C**) Genotype distribution of offspring from heterozygous (Flt1$^{+/In13pA}$) parental mice. (**D**) Schematic representation and sequencing results of the oligo(dT) primed RACE-PCR product from liver tissue cDNA, showing a read-through of 115 nucleotides in intron 13, encoding an alternative 32 amino acid sFlt1 tail, demonstrating correct alternative pre-mRNA processing in *Flt1*^{In13pA} mice. Flt1In13pA genomic mutations and the putative polyadenylation signal hexamer are depicted in red. (**E**) Western blot analysis of liver protein lysate showing complete knockout of flFlt1 and subsequent increase in sFlt1 (n=4-5 *Flt1*$^{+/+}$ and n=5 *Flt1*^{In13pA} mice). Data represent mean ± SD. Results were analyzed by two-tailed, unpaired Student t test. ****$p<0.0001$.

Supplemental Figure 3

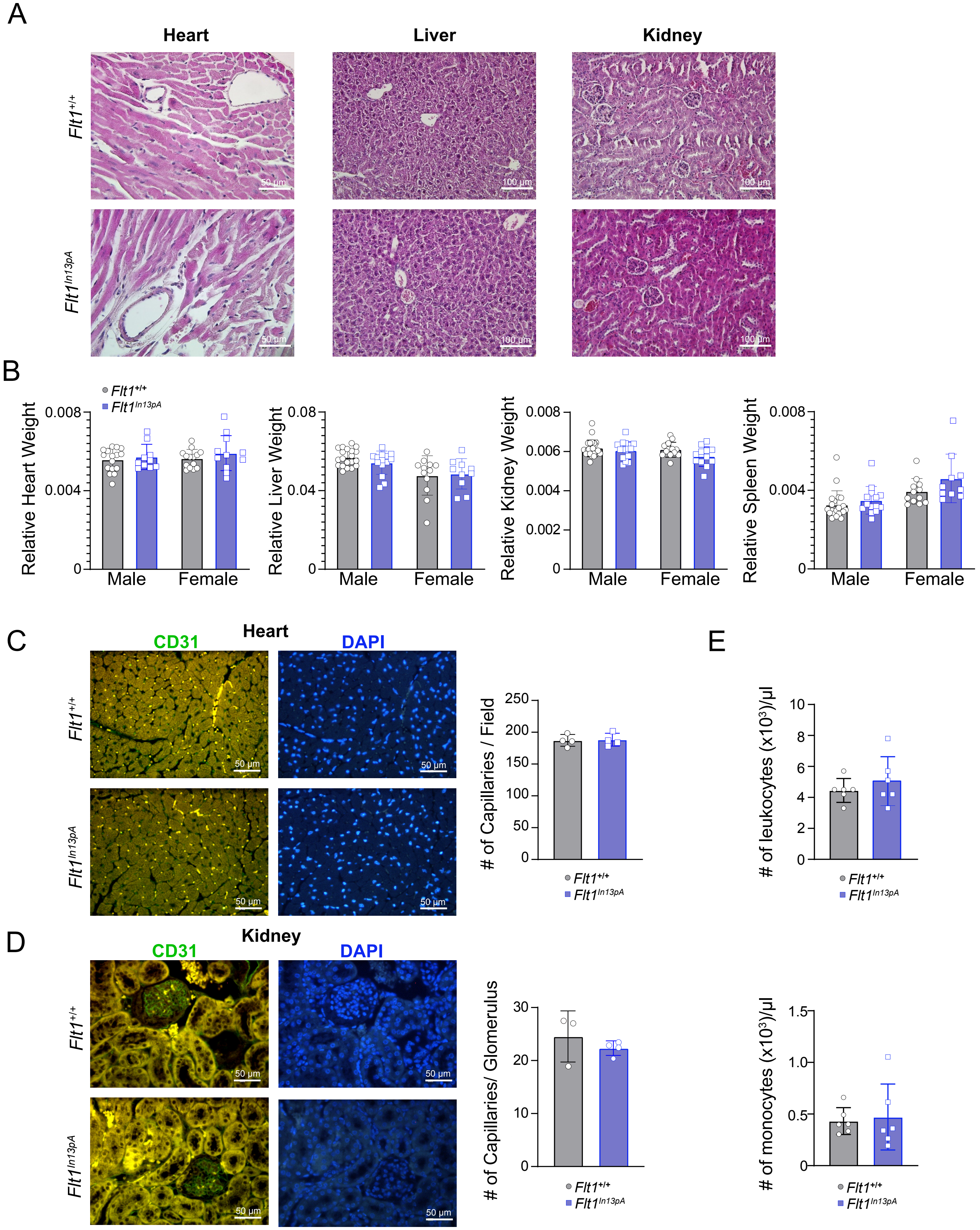

Supplemental Figure 3. (**A**) Tissues sections of *Flt1*$^{+/+}$ and *Flt1*^{In13pA} heart, liver and kidney stained with H&E; scale bars for heart, 50µm, and for liver and kidney, 100µm. (**B**) Weight of heart, liver, kidney, and spleen relative to total body weight in male and female 8-week-old *Flt1*$^{+/+}$ and *Flt1*^{In13pA} mice (n=10-23 *Flt1*$^{+/+}$ and n=10-15 *Flt1*^{In13pA} mice). (**C**) Representative immunofluorescence staining of CD31 and DAPI in heart and kidney tissue sections from *Flt1*$^{+/+}$ and *Flt1*^{In13pA} mice (scale bars, 50 µm). (**D**) Number of CD31−positive capillaries per microscopic field in tissue sections through the heart (n=4 mice per genotype) and kidney glomeruli (n=3 *Flt1*$^{+/+}$ and n=4 *Flt1*^{In13pA} mice) of 8-week-old male *Flt1*$^{+/+}$ and *Flt1*^{In13pA} mice. (**E**) Number of blood leukocytes and monocytes. Data represent mean ± SD. Results were analyzed by two-tailed, unpaired Student t−test.

Supplemental Figure 4

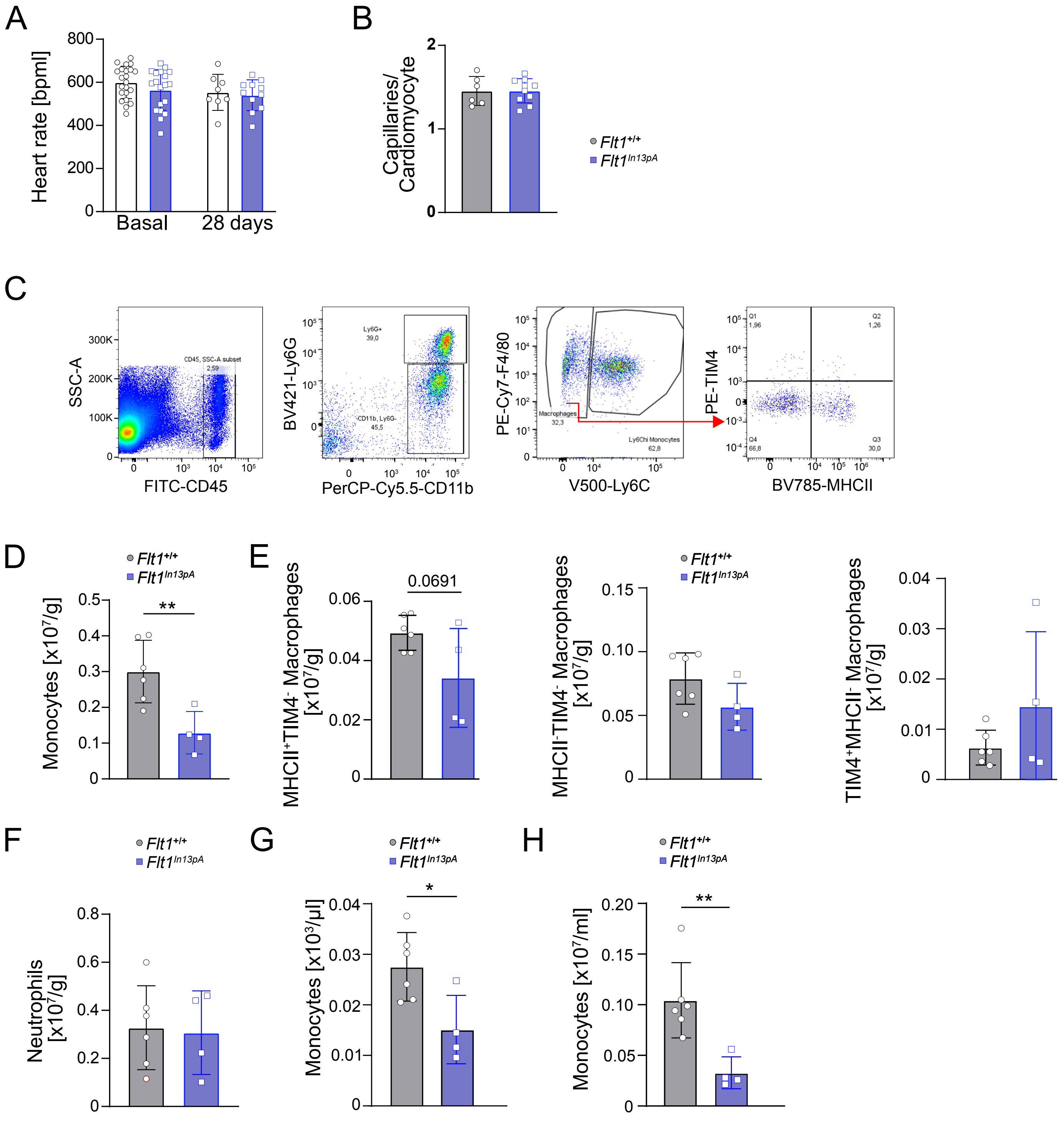

Supplemental Figure 4. (**A-E**) *Flt1*$^{+/+}$ and *Flt1*^{In13pA} mice were subjected to MI injury. (**A**) Heart rate before (n=22 Flt1$^{+/+}$ and n=19 Flt1^{In13pA} mice) and at day 28 post-MI (n=8 *Flt1*$^{+/+}$ and n=11 *Flt1*^{In13pA} mice). Data were analyzed by One way ANOVA followed by Tukey's post-hoc test. bpm – beats per minute. (**B**) Quantification of CD31^{+} capillaries relative to the number of cardiomyocytes in the remote myocardium (n=6 *Flt1*$^{+/+}$ and n=10 *Flt1*^{In13pA} mice) at day 28 after MI. Results were analyzed by a two-tailed, unpaired Student t-test. (**C**) Gating strategy to evaluate monocyte, macrophage, and neutrophil counts in the heart 3 days post-MI. (**D-G**) Quantification of the number of monocytes (**D, G, H**) and (**E**) MHCII^{+}, TIM4^{+} macrophages and (**F**) neutrophils in the infarcted myocardium (**D-F**), blood (**G**), and bone marrow (**H**) at day 3 post-MI (n=6 *Flt1*$^{+/+}$ and n=4 *Flt1*^{In13pA} mice), as assessed by flow cytometry. Results were analyzed by unpaired Student t-test. Data represent mean ± SD. *p<0.05, **p<0.01, ***p<0.001, ****p<0.0001.

Supplemental Figure 5

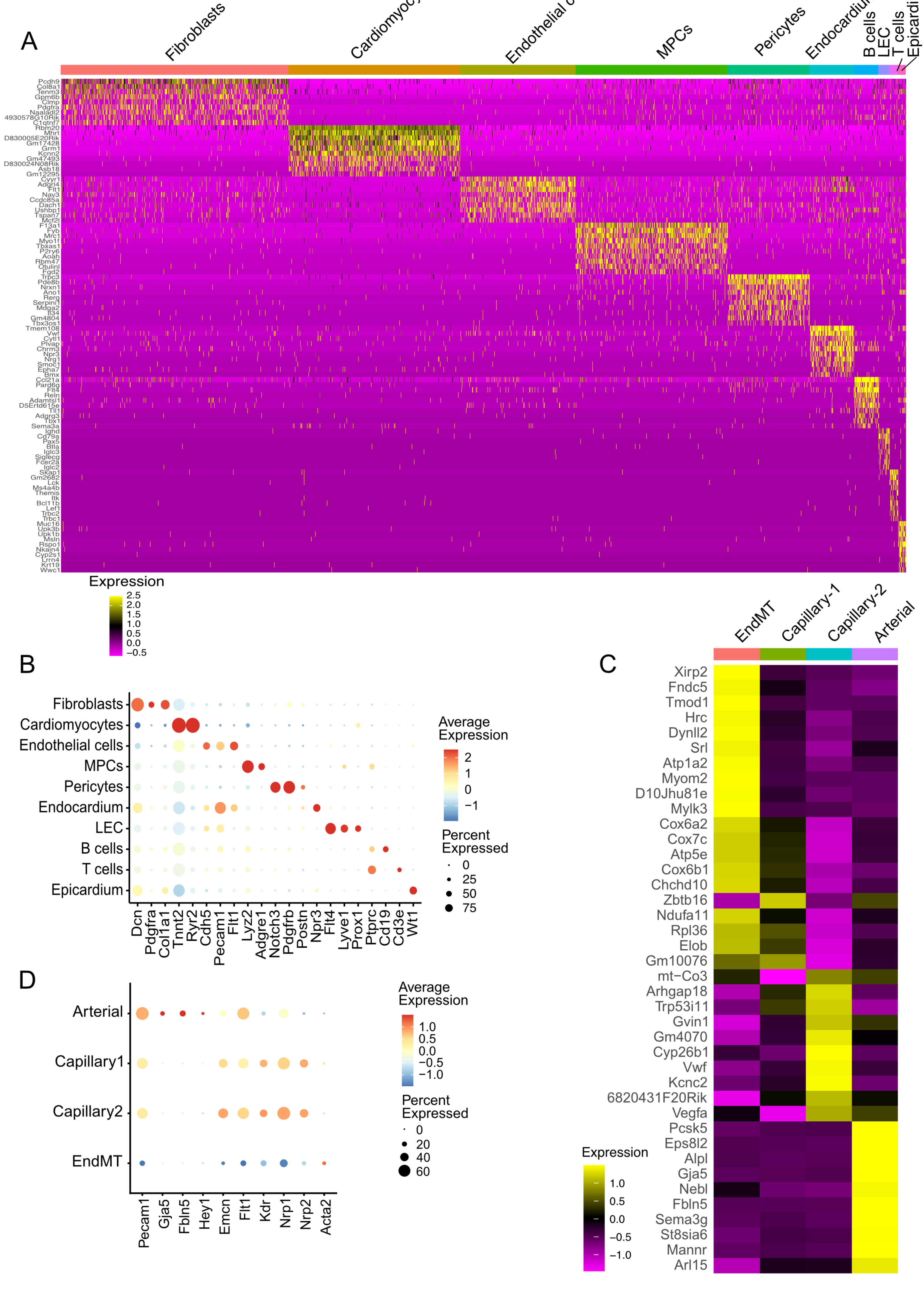

Supplemental Figure 5. Single-nuclei clustering analysis of hearts on day 28 after myocardial infarction. (**A**) Heat map depicting the average expression value of top 10 differentially expressed markers across different Seurat clusters. (**B**) Conventional marker genes used for the annotation of cell types. (**C**) Heat map depicting the average expression value of top 10 differentially expressed markers across endothelial cells sublcusters. (**D**) Conventional marker genes used for the annotation of endothelial cells subclusters.

Supplemental Figure 6

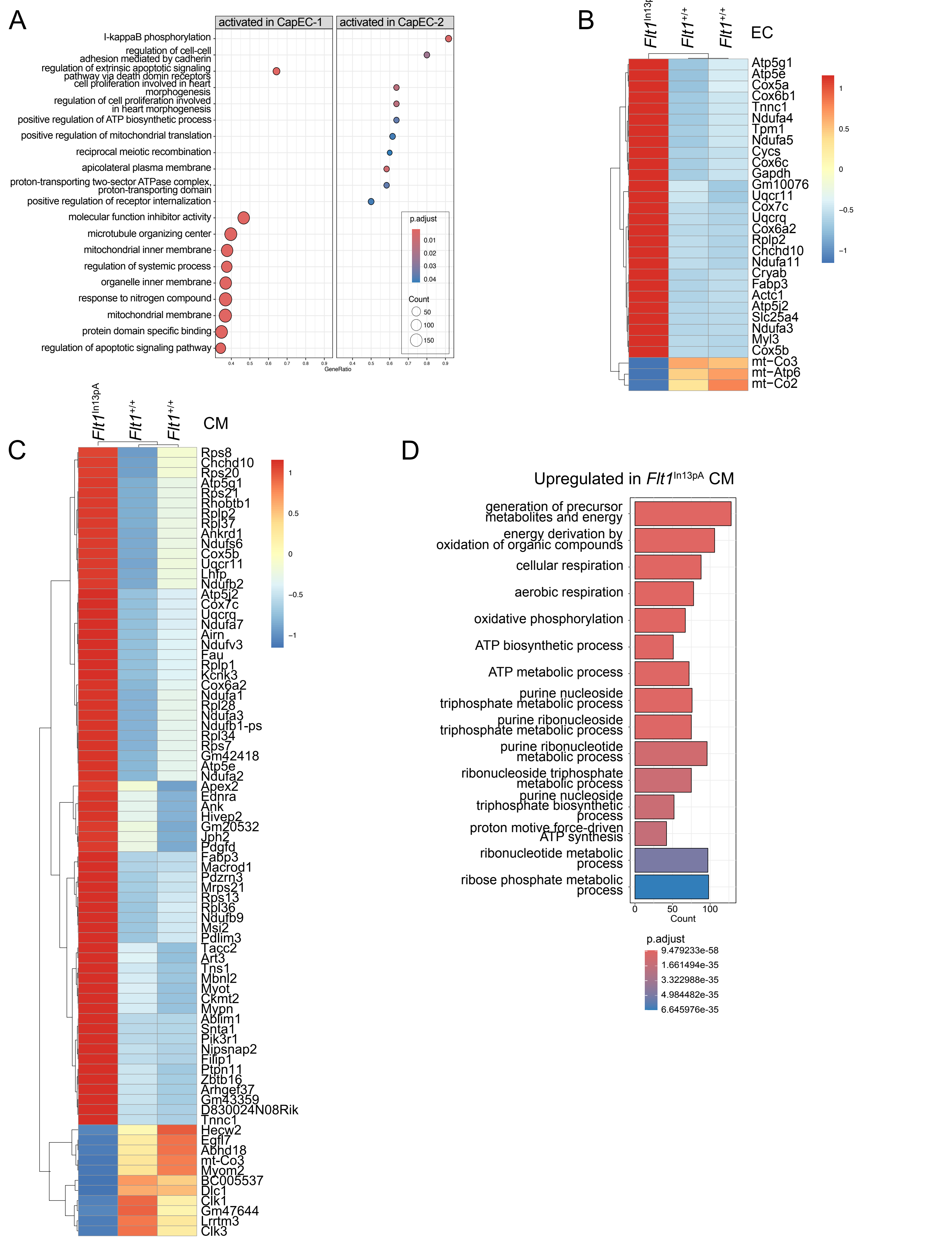

Supplemental Figure 6. (A) GO-term analysis of DEG upregulated in CapEC-1 and CapEC-2 clusters. (**B**) DEG between endothelial cells **(B)** and cardiomyocytes **(C)** in *Flt1*^{In13pA} in comparison to *Flt1*$^{+/+}$ hearts. **(D)** GO-term analysis of DEG upregulated in cardiomyocates (CM) from *Flt1*^{In13pA} in comparison to *Flt1*$^{+/+}$ hearts.

Supplemental Figure 7

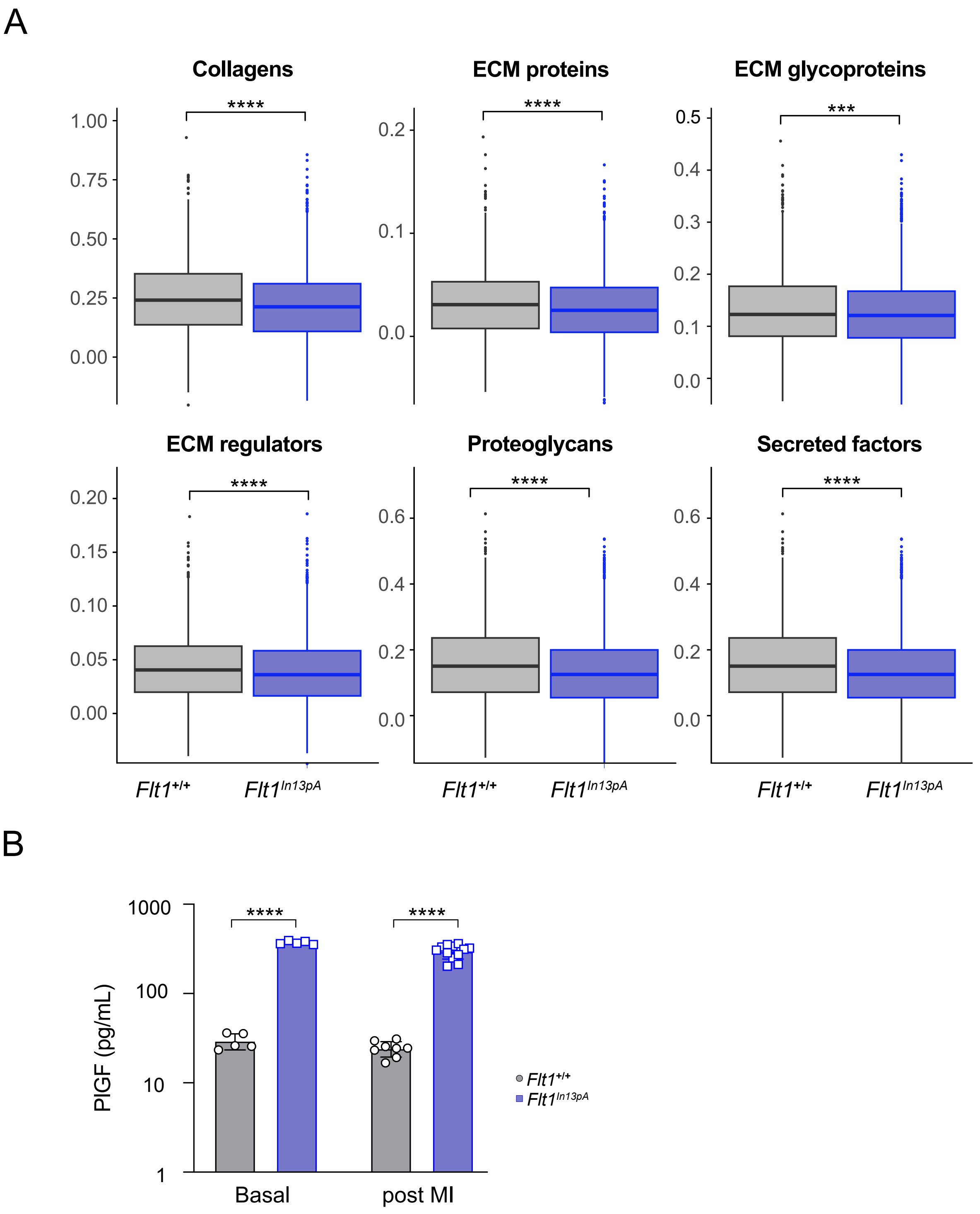

Supplemental Figure 7. (**A**) Matrisome scores in fibroblastst from *Flt1*$^{+/+}$ and *Flt1*^{In13pA} hearts on day 28 after MI. The center lines indicate the median and box limits represent interquartile ranges (IQR). Significance was calculated using the t.test method from the SeuratExtend package. (**B**) PlGF serum levels before (n=5 *Flt1*$^{+/+}$ and n=5 *Flt1*^{In13pA} mice) and at day 28 post-MI (n=8 *Flt1*$^{+/+}$ and n=11 *Flt1*^{In13pA} mice). Results were analyzed by unpaired Student t−test. Data represent mean ± SD. ***p<0.001, ****p<0.0001.

Supplemental Figure 8

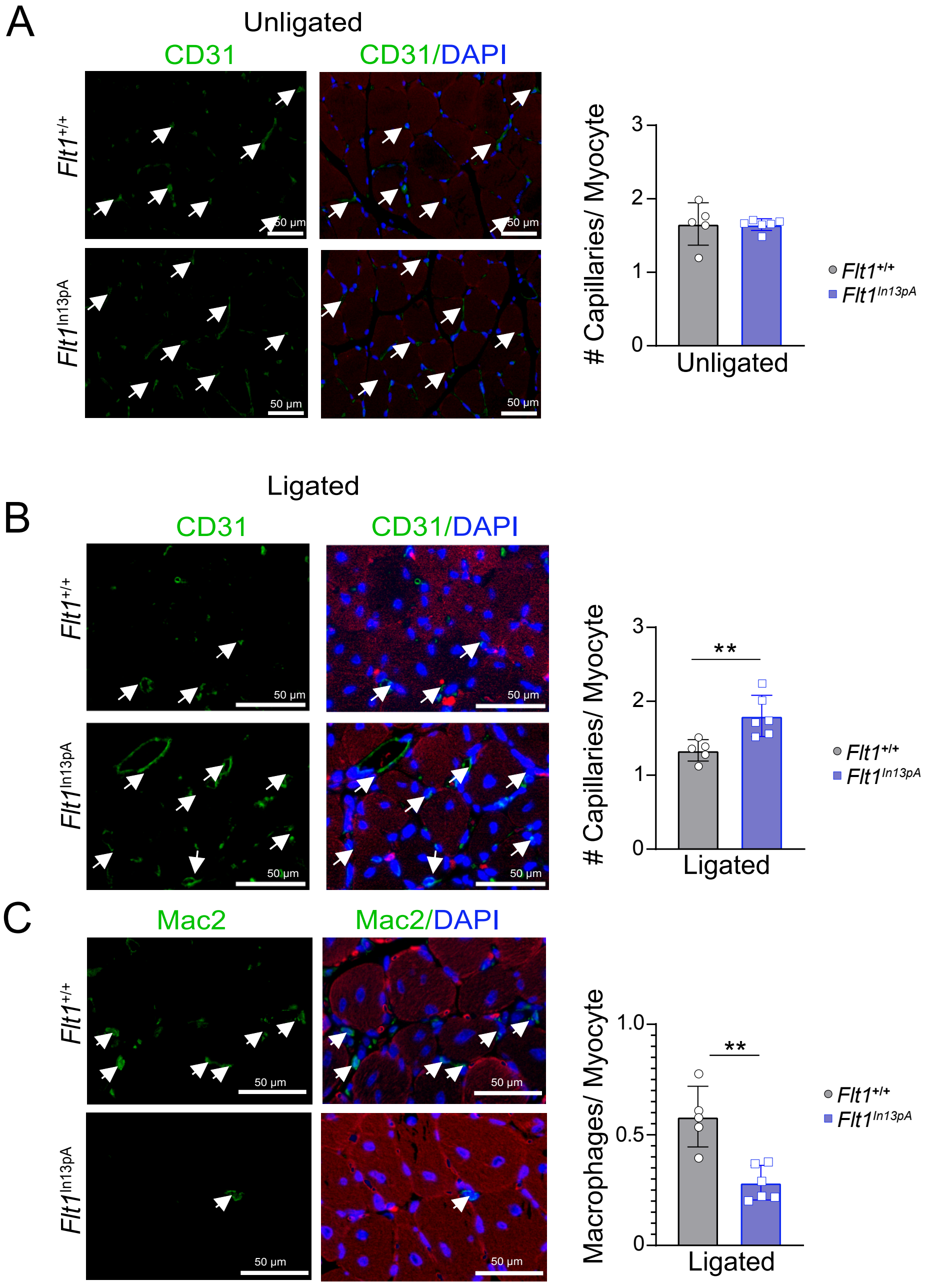

Supplementary Figure 8. In the hindlimb ischemia model, *Flt1*$^{+/+}$ (n=5) and *Flt1*^{In13pA} (n=6) mice were studied at 14 days after injury. Representative immunofluorescence staining and quantification of CD31^{+} capillaries in (**A**) unligated gastrocnemius muscle, and in the (**B**) ligated gastrocnemius muscle. (**C**) Representative immunofluorescence staining and quantification of Mac2^{+} monocytes/macrophages in the ischemic gastrocnemius muscle. Scale bars, 50 µm. Data represent mean ± SD. Results were analyzed by two-tailed, unpaired Student t-test. **p<0.01.

Supplemental Figure 9

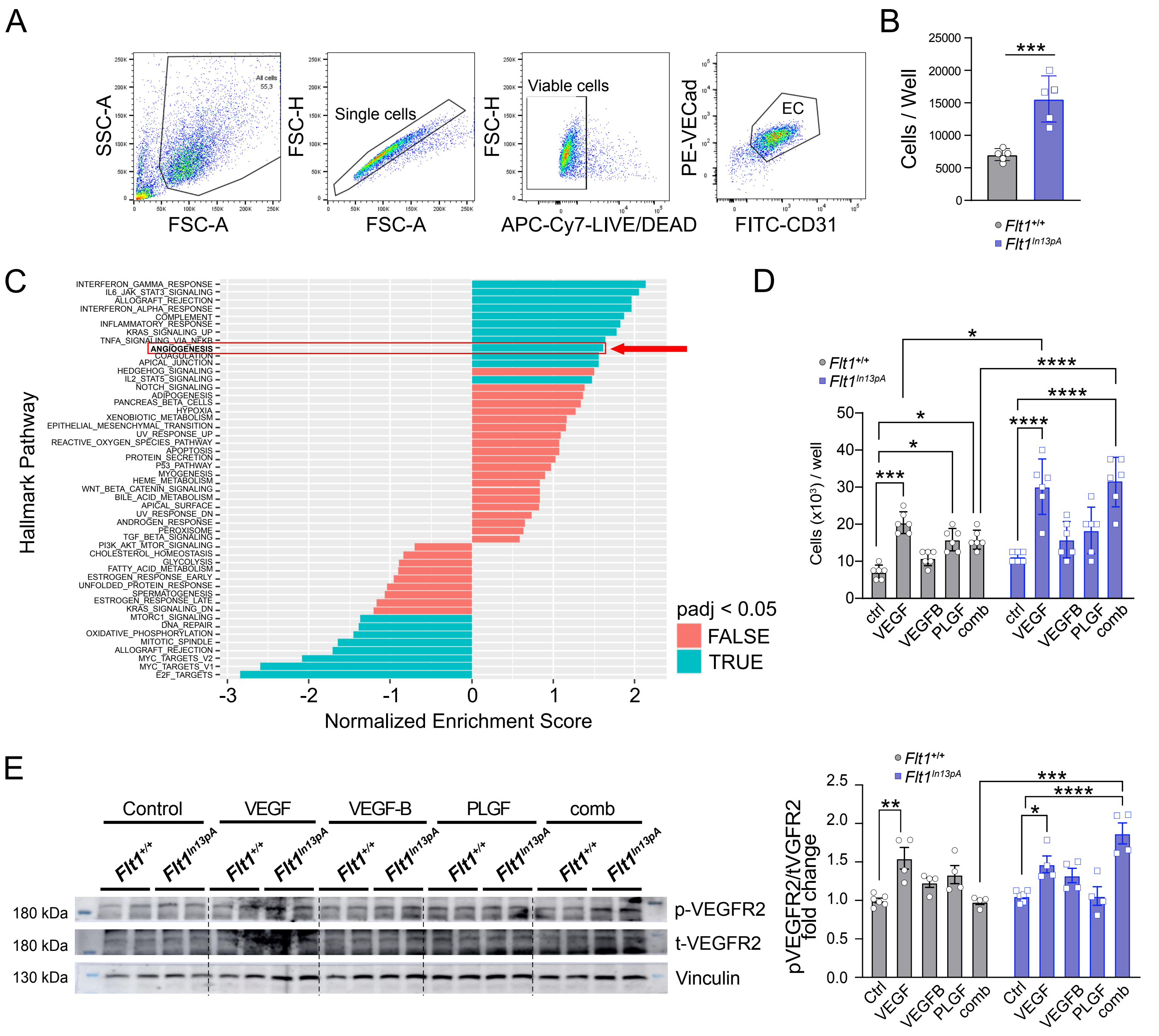

Supplemental Figure 9. (**A**) Gating strategy to evaluate the % of CD31⁺CD144⁺ primary mouse lung endothelial cells amongst total live cells by flow cytometry. (**B**) Endothelial cell proliferation. *Flt1*$^{+/+}$ and *Flt1*^{In13pA} mouse lung endothelial cells were grown for 48 h, detached and counted (independent endothelial cell isolations from n = 5 mice per genotype). Results were analyzed by a two-tailed, unpaired Student t-test. (**C**) Waterfall plot against MSigDB hallmark gene sets with a significant cutoff determined by padj< 0.05, depicting differential pathway enrichment in *Flt1*$^{+/+}$ and *Flt1*^{In13pA} lung endothelial cells exposed to hypoxia over 24 h (n=3 experiments performed with pooled endothelial cells isolated from n=8 mice). (**D**) Endothelial cell proliferation. *Flt1*$^{+/+}$ and *Flt1*^{In13pA} mouse lung endothelial cells were left untreated (Ctrl) or treated with VEGF, VEGFB, PIGF, or a combination of these cytokines (comb) and grown for 48 h, detached and counted. Results were analyzed using one-way ANOVA followed by Tukey's post-hoc test. (**E**) Phosphorylated VEGFR2 (p-VEGFR2), total VEGFR2 (t-VEGFR2) and Vinculin was analyzed in lysates of mouse lung endothelial cells left untreated (control) or treated with VEGF, VEGFB, PIGF, or a combination of these cytokines (comb) for 5 min by Western blotting (n=6 per genotype). Results were analyzed by One−way ANOVA followed by Tukey's post-hoc test. Data represent mean ± SD. *p<0.05, ***p<0.001, ****p<0.0001.

Supplemental Figure 10

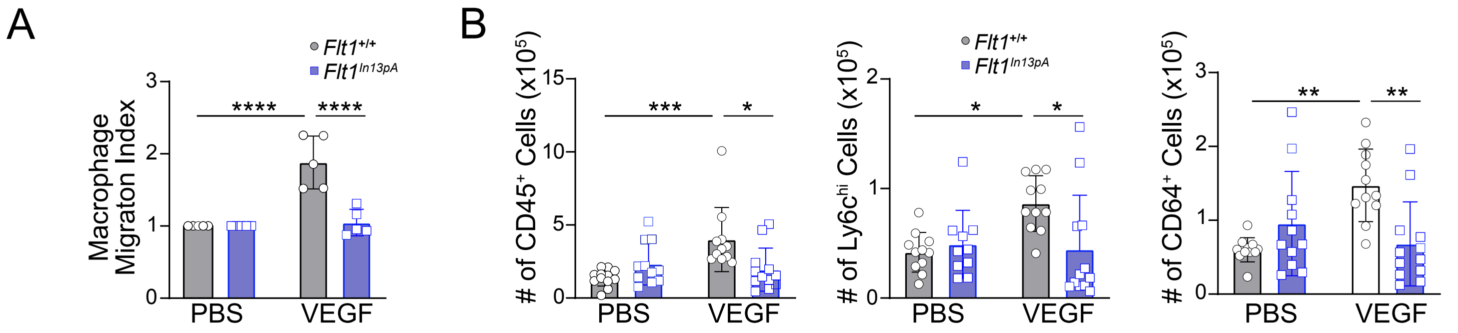

Supplemental Figure 10. (**A**) Chemotaxis of *Flt1*$^{+/+}$ and *Flt1*^{In13pA} bone marrow-derived macrophages towards VEGF compared to vehicle (independent BMDM cultures from n=5 mice per genotype). (**B**) Number of CD45^{+} leukocytes, Ly6C^{hi} monocytes, and of CD64^{+} macrophages in the air pouch of *Flt1*$^{+/+}$ and *Flt1*^{In13p} mice, injected with PBS or VEGF and analyzed by flow cytometry after 24 h (n=10-12 mice per genotype). Data were pooled from 3 independent experiments. Results were analyzed by One–way ANOVA followed by Tukey's post-hoc test. Data represent mean ± SD. *p<0.05, **p<0.01, ***p<0.001, ****p<0.0001.
