# Supplementary material for: Redirecting full-length FLT1 expression towards its soluble isoform by targeting alternative polyadenylation promotes postischaemic angiogenesis
Source: Cardiovasc Res. 2026 Oct 5;122(15):2160–76. doi: 10.1093/cvr/cvag198 (PMC13742059; doi:10.1093/cvr/cvag198)
Supplement: Supplementary file 1 [file cvag198_supplementary_data.zip › Uncropped Western Blot images_Supplementary Figures.pdf]

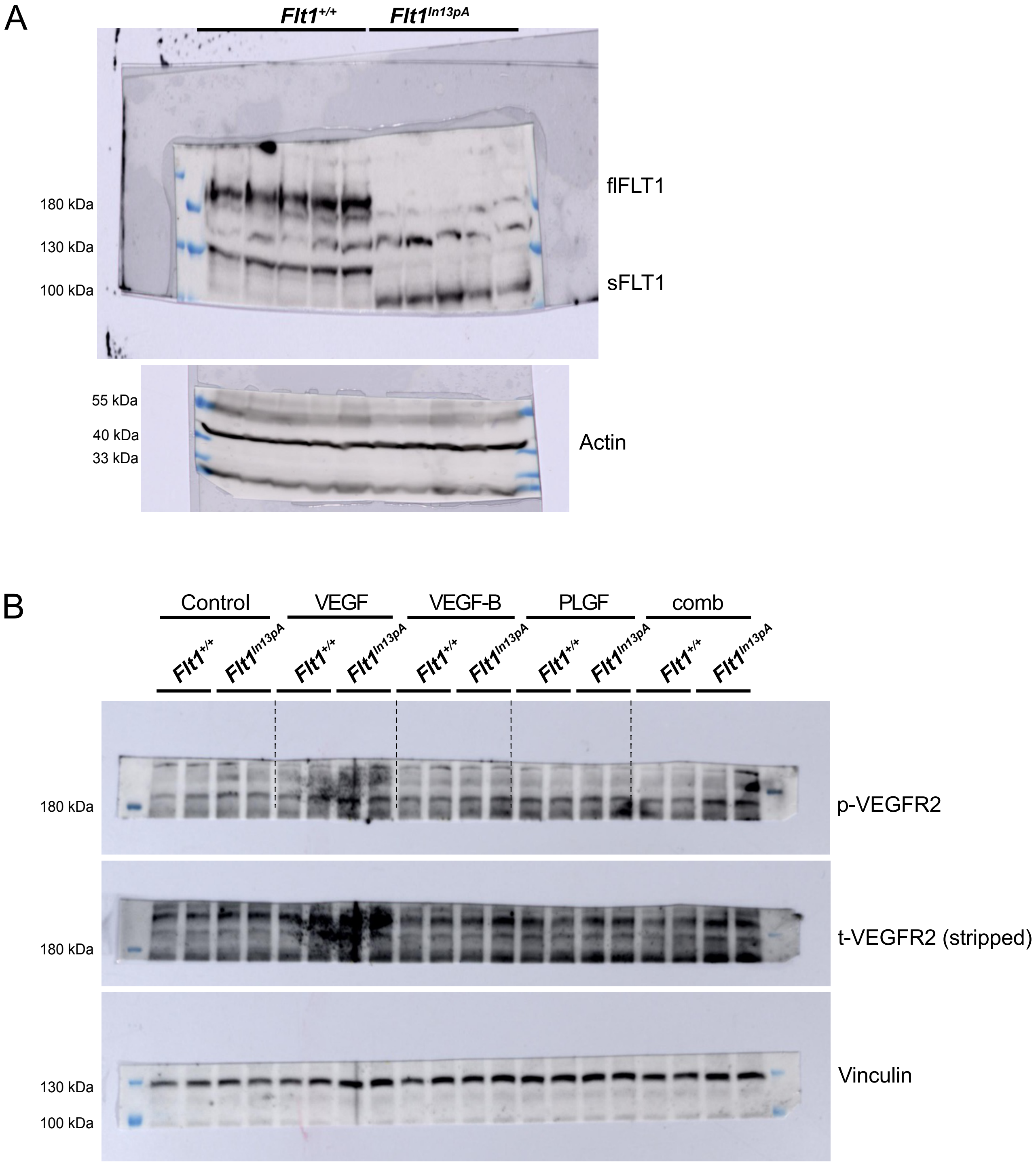

Supplemental Material 2. (**A**) Uncropped Western Blot images of the blots shown in Supplementary Figure 2E. (**B**) Uncropped Western Blot images of the blots shown in Supplementary Figure 9E. Molecular mass marker positions are indicated in kilodaltons (kDa).
